# *NOTCH2NLC* GGC intermediate repeat with serine induces hypermyelination and early Parkinson’s disease-like phenotypes in mice

**DOI:** 10.1186/s13024-024-00780-2

**Published:** 2024-11-28

**Authors:** Haitao Tu, Xin Yi Yeo, Zhi-Wei Zhang, Wei Zhou, Jayne Yi Tan, Li Chi, Sook-Yoong Chia, Zhihong Li, Aik Yong Sim, Brijesh Kumar Singh, Dongrui Ma, Zhidong Zhou, Isabelle Bonne, Shuo-Chien Ling, Adeline S.L. Ng, Sangyong Jung, Eng-King Tan, Li Zeng

**Affiliations:** 1 Neural Stem Cell Research Lab, Research Department, National Neuroscience Institute, Singapore, 308433 Singapore; 2 Department of Psychological Medicine, Yong Loo Lin School of Medicine, National University of Singapore, Singapore, 119228 Singapore; 3 Research Department, National Neuroscience Institute, Singapore General Hospital (SGH) Campus, Singapore, 169856 Singapore; 4 Department of Neurology, National Neuroscience Institute, Singapore, 308433 Singapore; 5 Hospital of Stomatology, Guanghua School of Stomatology, Guangdong Provincial Key Laboratory of Stomatology, Institute of Stomatology, Sun Yat-Sen University, Guangzhou, Guangdong 510080 China; 6 Electron Microscopy Unit, Yong Loo Lin School of Medicine, National University of Singapore, Singapore, 117549 Singapore; 7 Laboratory of Hormonal Regulation, Cardiovascular and Metabolic Disorders, Duke-NUS Medical School, Singapore, 169857 Singapore; 8 Department of Neurology, Singapore General Hospital, Singapore, 169609 Singapore; 9 Neuroscience & Behavioural Disorders Program, DUKE-NUS Graduate Medical School, Singapore, 169857 Singapore; 10 Department of Microbiology and Immunology, Yong Loo Lin School of Medicine, National University of Singapore, Singapore, 117545 Singapore; 11 Immunology Translational Research Programme, Life Sciences Institute, National University of Singapore, Singapore, 117456 Singapore; 12 Department of Physiology, Yong Loo Lin School of Medicine, National University of Singapore, Singapore, 119077 Singapore; 13 Department of Medical Science, College of Medicine, CHA University, Seongnam, 13488 Republic of Korea; 14 Centre for Molecular Neuropathology, Lee Kong Chian School of Medicine, Nanyang Technology University, Singapore, Novena Campus, 308232 Singapore

**Keywords:** *NOTCH2NLC*, GGC repeat expansion, Intermediate repeat, AGC interruption, Mitochondrial dysfunction, Hyperexcitability, Hypermyelination, Early Parkinson’s disease

## Abstract

**Background:**

The expansion of GGC repeats (typically exceeding 60 repeats) in the 5’ untranslated region (UTR) of the *NOTCH2NLC* gene (N2C) is linked to N2C-related repeat expansion disorders (NREDs), such as neuronal intranuclear inclusion disease (NIID), frontotemporal dementia (FTD), essential tremor (ET), and Parkinson’s disease (PD). These disorders share common clinical manifestations, including parkinsonism, dementia, seizures, and muscle weakness. Intermediate repeat sizes ranging from 40 to 60 GGC repeats, particularly those with AGC-encoded serine insertions, have been reported to be associated with PD; however, the functional implications of these intermediate repeats with serine insertion remain unexplored.

**Methods:**

Here, we utilized cellular models harbouring different sizes of N2C variant 2 (N2C2) GGC repeat expansion and CRISPR-Cas9 engineered transgenic mouse models carrying N2C2 GGC intermediate repeats with and without serine insertion to elucidate the underlying pathophysiology associated with N2C intermediate repeat with serine insertion in NREDs.

**Results:**

Our findings revealed that the N2C2 GGC intermediate repeat with serine insertion (32G13S) led to mitochondrial dysfunction and cell death in vitro. The neurotoxicity was influenced by the length of the repeat and was exacerbated by the presence of the serine insertion. In 12-month-old transgenic mice, 32G13S intensified intranuclear aggregation and exhibited early PD-like characteristics, including the formation of α-synuclein fibers in the midbrain and the loss of tyrosine hydroxylase (TH)-positive neurons in both the cortex and striatum. Additionally, 32G13S induced neuronal hyperexcitability and caused locomotor behavioural impairments. Transcriptomic analysis of the mouse cortex indicated dysregulation in calcium signaling and MAPK signaling pathways, both of which are critical for mitochondrial function. Notably, genes associated with myelin sheath components, including *MBP* and *MOG*, were dysregulated in the 32G13S mouse. Further investigations using immunostaining and transmission electron microscopy revealed that the N2C intermediate repeat with serine induced mitochondrial dysfunction-related hypermyelination in the cortex.

**Conclusions:**

Our in vitro and in vivo investigations provide the first evidence that the N2C-GGC intermediate repeat with serine promotes intranuclear aggregation of N2C, leading to mitochondrial dysfunction-associated hypermyelination and neuronal hyperexcitability. These changes contribute to motor deficits in early PD-like neurodegeneration in NREDs.

**Supplementary Information:**

The online version contains supplementary material available at 10.1186/s13024-024-00780-2.

## Background

*NOTCH2NLC* (N2C) with GGC repeat expansions has been identified as a causative factor in neuronal intranuclear inclusion disease (NIID), a neurodegenerative disorder characterized by ataxia, dementia, parkinsonism, and neuropathy [1–3]. Pathogenic N2C-GGC repeat expansions in NIID are usually more than 60 repeats and many patients have more than 130 repeats. Normal repeat expansions are typically less than 30 repeats and repeats between 40 and 60 are in the intermediate range, and its pathogenicity is unclear. Pathologically, the condition is marked by eosinophilic intranuclear inclusions within neurons and glial cells [1–3]. N2C expansions are also found in patients diagnosed with frontotemporal disorders (FTD) [4], Parkinson’s disease (PD) [5, 6], essential tremor (ET) [7, 8], and other conditions, sharing common neuroimaging, histopathological features, and clinical manifestations [9]. Several proposed mechanisms for N2C-GGC repeat expansion-induced pathogenesis include loss-of-function due to GGC expansion-induced transcription inhibition, toxic RNA gain-of-function, and expanded protein gain-of-function [10–12]. Recent studies suggest that a polyglycine (polyG) encoding protein from N2C-GGC repeat promotes neurotoxicity [13–15]. Despite these insights, the precise molecular mechanisms underlying disease pathogenesis remain elusive.

N2C is among the four human-specific *NOTCH2*-related genes (*NOTCH2NLA*, *NOTCH2NLB*, *NOTCH2NLC*, and *NOTCH2NLR*) within the human genome. N2C, highly expressed in the brain, plays a pivotal role in the evolutionary expansion and development of the human brain [16]. Trinucleotide GGC expanded repeats in the 5’ untranslated region (UTR) of the *NOTCH2NLC* gene were initially reported to be the cause of NIID [2, 3]. Skin biopsy in NIID patients revealed eosinophilic intranuclear inclusions [17]. N2C has two isoforms: isoform 1 (N2C1), where the GGC repeat expansion is located in the 5’-UTR, and isoform 2 (N2C2), where the GGC repeat is within the protein-coding region. The GGC repeat expansion in the 5’-UTR of N2C1 forms an upstream open reading frame and has been shown to translate into a pathogenic polyG-containing protein (uN2C-polyG) in NIID [2, 13, 15]. Additionally, studies revealed that N2C2 comprises 10–16% of total N2C transcripts [15, 18]. The GGC repeat expansion in N2C2 also encodes a pathogenic polyglycine-containing protein (N2C2-polyG), recently shown to respond to osmotic stress in vitro [18]. However, the pathophysiologic role of N2C2-polyG in neurodegeneration has not been explored.

We have previously identified pathogenic N2C-GGC repeat expansions in sporadic ET patients carrying more than 80 GGC repeats [7]. Notably, approximately 8% of these cases exhibited AGC insertion within the N2C-GGC repeat expansion. Separately, we also detected N2C-GGC repeat expansions in patients with typical PD clinical features harbouring more than 40 repeat expansions [5]. Several PD patients carry the intermediate N2C GGC expansions, ranging from 41 to 64 repeats. Interestingly, the frequency of AGC insertion in these intermediate repeats was three times higher in PD patients with N2C GGC repeat expansions than in patients with NIID (25% ± 12% vs. 8% ± 8%). This suggests that AGC insertion within the GGC expansion and the repeat size may contribute to parkinsonism. Our hypothesis posits that intermediate GGC repeat with AGC insertion may expedite N2C-GGC-associated neurotoxicity in *NOTCH2NLC*-related repeat expansion disorders (NREDs).

Recent studies using N2C-polyG transgenic animal models expressing large pathogenic expansions (> 60 repeats) have been reported. By comparing the uN2C-100G versus 13G repeat expansion, Yu et al. [19] found progressive retinal degeneration, locomotor impairment, reduced lifespan, and mitochondrial dysregulation in Drosophila models. Meanwhile, several transgenic or adeno-associated virus (AAV)-infected N2C-polyG mouse models showed reduced lifespan, mitochondrial dysfunction, cardiac dysfunction, muscle degeneration, motor and cognitive impairment, and dysregulated microglia in high repeat uN2C-polyG [13, 15, 20–22] (summarized in Supplementary Table 1). In 2023, Fan et al. [23] generated several lines of patient induced pluripotent stem cells (iPSCs)-derived cerebral organoids (> 90G) and revealed that uN2C-polyG may induce nucleolar stress and impair ribosome biogenesis by single-cell transcriptomics. However, all these models focus on the high GGC repeat in N2C1, the roles of intermediate repeat and AGC insertion have not been studied. Moreover, a genetic animal model for intermediate repeat and AGC insertion particularly in N2C2 is lacking.

Here, we generated a series of transgenic mouse models in which N2C2 is expressed with control (GGC_30_, or N2C-30G), intermediate repeat (GGC_45_, or N2C-45G), or intermediate repeat with average percentage of AGC (encoding serine) insertion (GGC_32_AGC_13_, or N2C-32G12S) found in N2C-carrying PD patients. We showed that the intermediate repeat with serine insertion promotes intranuclear inclusion formation, impairs mitochondrial function, and induces neuronal death in vitro. N2C-32G13S mice showed more pronounced intranuclear protein aggregation, mitochondrial dysfunction, hyperexcitability, and motor deficits compared to N2C-45G and N2C-30G mice. Additionally, we observed α-synuclein fiber formation in the 32G13S mouse midbrain but not the striatum, and tyrosine hydroxylase reduction in both the striatum and cortex but not the midbrain, implying an early PD-like pathological feature in mice. Further, the transcriptomic analysis revealed dysregulation of mitochondrial-related pathways and myelin sheath component genes, which induced hypermyelination in the 32G13S mouse cortex. Together, our studies provide the first evidence that N2C-polyG intermediate repeat with serine promotes mitochondrial impairment and hypermyelination that is associated with motor disability, compatible with clinical observations in early PD patients.

## Methods

### Patient samples

Human blood was collected from patients from the National Neuroscience Institute of Singapore (NNI), who were recruited for this study with fully informed written consent. All methods and protocols used in this study were performed following the institutional and relevant guidelines and regulations. The clinical diagnosis and pathological features were carefully examined by clinicians and pathologists from NNI. This study was also approved by the Institutional Review Board of NNI (2017/2602).

### iPSC reprogramming and neuron differentiation

Peripheral blood mononuclear cells (PBMCs) were collected from patient blood and were reprogrammed into iPSCs using CytoTune-iPS Sendai Reprogramming Kit following the manufacturer’s instruction (Thermo Fisher, #A16517). Patient PBMC-derived iPSCs were further differentiated into neurons. Briefly, iPSCs were dissociated using Accutase and seeded in ultra-low attachment culture wells with mTeSR medium (STEMCELL, #85850) and Y-27,632 ROCK inhibitor (STEMCELL, #72304) for 48 h. The medium was changed to DUAL SMAD medium (NEAA, Glutamax, N2, B27, LDN, and SB431542) supplemented with Y-27,632 for neural progenitor cells (NPCs) differentiation. The medium was changed every two days. On day 5, embryo bodies were formed and seeded onto Matrigel-coated wells (Corning, #354277). DUAL SMAD medium was changed daily. On day 11, the attached embryo bodies were dissociated using Accutase. The cells were then seeded on Matrigel-coated wells with differentiation medium (NEAA, Glutamatx, B27, BDNF, GDNF, ascorbic acid, and dbcAMP) with daily changes for 7 days. Neurons were then fixed in 4% paraformaldehyde for further analysis.

### CRISPR/Cas9-mediated genomic correction of *NOTCH2NLC* GGC repeat expansion in patient iPSCs

*NOTCH2NLC* patient iPSC was cultured in mTeSR Plus medium (Stemcell technologies) on tissue culture plates coated with Geltrex (Thermo fisher scientific). To generate an isogenic cell line, 80% confluent iPSCs were digested with Accutase (Thermo fisher scientific) to get a single cell suspension and subjected to electroporation. 2.0E + 5 iPSC cells were incubated with 2 µg of Cas9 mRNA, 0.3 µg of gRNA mRNA (gRNA sequence: AGGCTCAGGCCCTGGCGCTA CGG), and 1 µl of 10 µM single strand oligo donor (IDTDNA) to form ribonucleoprotein. Electroporation was performed using Neon Transfection System 10 µL Kit (Thermo Fisher Scientific). Electroporation conditions were optimized at 850 V, 30 ms, and 2 pulses. The iPSC cells were immediately plated onto Geltrex-coated plates in mTesR Plus medium containing 10 µM ROCK inhibitor Y27632. Cells were counted after 48 h of incubation, followed by serial dilution onto 96 well plates with a cell density of 1 cell per plate. After clonal expansion for 7–10 days, cells from individual wells were harvested, followed by genomic DNA extraction, repeat-primed PCR, and GGC repeat size determination [5]. The gRNA was designed using the IDT online gRNA Design Tool. The top 5 potential off-targets were verified using PCR and sanger sequencing. No off-target effect was detected, including off-targets on *NOTCH2NLA* and *NOTCH2NLB*.

### Generation of N2C-polyG transgenic mice

The humanized N2C2-30G, -45G, and − 32G13S transgenic mice used in this study were generated by Cyagen Inc. Briefly, the N2C2-polyG expression cassettes consist of a human *Nes* gene promoter, which specifically expressed in CNS progenitor cells and early neural crest cells [24, 25], synthetic N2C2-30G, -45G, or -32G13S, and a C-terminal HA tag. The N2C2-polyG expression cassettes are flanked by two mouse *ROSA26* DNA fragment arms. The gRNA CTCCAGTCTTTCTAGAAGATGGG was used to target the mouse genome at the *ROSA26* locus. The nuclease mRNA and N2C2-polyG expression vector were co-injected into fertilized mouse (C57/6 N) eggs, followed by the implantation of eggs into surrogate mothers to obtain offspring. Primers 5’ CACTTGCTCTCCCAAAGTCGCTC 3’ and 5’ ATACTCCGAGGCGGATCACAA 3’ were used to detect non-transgenic (NTg) mice. Primers 5’ GGCAGACTGGTGACTTCACTTT 3’ and 5’ CTTTATTAGCCAGAAGTCAGATGC 3’ were used to detect transgenic mice. Animals were maintained under institutional guidelines, and all protocols were approved by the Institutional Animal Care and Use Committee (IACUC) of the National Neuroscience Institute (NNI) and Tan Tk Seng Hospital. Mice were maintained in a specific-pathogen-free facility and were exposed to a 12-hour light/dark cycle. Food and water were continuously available. Both sexes and heterozygous mice were used for all studies.

### Whole-cell patch clamp recording in acute brain slices

Experiments were performed on acute brain slices prepared from humanized N2C-30G, -45G, or -32G13S transgenic mice. Mice were anaesthetized and trans-cardially perfused with high sucrose, ice-cold oxygenated slicing solution before the brain was rapidly removed after decapitation and placed in a fresh aliquot of the same ice-cold oxygenated solution. The slicing solution is a high sucrose variant of the artificial cerebrospinal fluid (ACSF) containing the following (in mM): 185 sucrose, 2.5 KCl, 1.2 NaH_2_PO_4_, 25 NaHCO_3_, 25 glucose, 10 MgSO_4_, and 0.5 CaCl_2_, pH 7.4, 300 mOsm, and 95% O_2_/5% CO_2_. Coronal slices of 250 μm thickness were made with a fully automated vibrating blade microtome (LT1200S, Leica Biosystems) and transferred immediately into an incubation chamber filled with ACSF containing the following (in mM): 124 NaCl, 2.5 KCl, 1.2 NaH_2_PO_4_, 24 NaHCO_3_, 12.5 glucose, 4 MgSO_4_, and 2 CaCl_2_, pH 7.4, 300 mOsm, equilibrated with 95% O_2_/5% CO_2_. Slices were allowed to recover at 32^o^C for 30 min and then maintained at room temperature for at least 30 min before use. Whole-cell patch-clamp recordings were performed on the prefrontal cortex neurons visualized using the ORCA-Flash4.0 LT3 Digital CMOS camera (C11440-42U40, Hamamatsu Photonics) and monitor. Pipettes used for recordings were pulled from borosilicate glass capillaries with filament (length 100 mm, outer diameter, 1.5 mm, inner diameter 0.84 mm; 1B150F-4, WPI) with the P-1000 micropipette puller (Sutter Instrument). Patch pipettes (5–7 MΩ) were filled with an internal solution containing (in mM) 130 K-gluconate, 5 NaCl, 11 KCl, 1 MgCl_2_, 10 HEPES, 0.1 EGTA, 2 Mg-ATP, 0.3 Na-GTP and 5 Na_2_-phosphocreatine (pH 7.3 with KOH; 295 mOsm), for recording. Access resistance, membrane resistance, and membrane capacitance were monitored during the experiment to ensure the stability and the health of cells. Action potentials are evoked by injection depolarizing current pulses in current-clamp mode. Signals were obtained with a 50 µs sampling rate (20 kHz signals) amplified with EPC 10 USB (HEKA Elektronic, Germany) and recorded with the accompanying PATCHMASTER (HEKA Elektronic, Germany) software. Data were analyzed using AxoGraph Ver 1.8.0 (AxoGraph Company) and ported to GraphPad Prism for statistical analysis.

### Bulk RNA sequencing and bioinformatic analysis

Cortex tissues from 12-month-old N2C-polyG mice were used for total RNA extraction. One microgram of RNA from each mouse cortex was used for directional RNA library preparation (poly A enrichment) and bulk RNA sequencing by NovogeneAIT using the NovaSeq PE150 platform at 150 bp 40 million reads. The raw differentially expressed gene list was further filtered with adjusted *P* value < 0.01 and fold change < 0.67 or > 1.5. The shortlisted genes were further analyzed for Gene Ontology enrichment and KEGG pathways analysis by the DAVID functional annotation bioinformatics analysis platform. Bioinformatics graphs were plotted by https://www.bioinformatics.com.cn, an online platform for data analysis and visualization [26].

### Transmission electron microscopy (TEM)

Mouse was intraperitoneally anaesthetized with ketamine/xylazine (100/10 mixture; 0.1 mg/g body weight) and perfused transcardially with 0.1 M phosphate-buffered saline (PBS), followed by 4% paraformaldehyde (PFA) and 0.5% glutaraldehyde in PBS. The mouse brain was isolated and sagittally cut into two halves. The left hemisphere of the brain was cut in the coronal plane at 0.3 mm from the bregma [27]. A 1–2 mm cube of cortical tissue beside the corpus callosum was isolated [28] and was fixed with a solution containing 4% paraformaldehyde and 2% glutaraldehyde in PBS overnight at 4^o^C. After washing three times with PBS, the sample was post-fixed for 1 h at room temperature with a mixture of potassium ferrocyanide-osmium tetroxide solution. The tissue sample was dehydrated in a graded series of ethanol and then infiltrated and embedded in Araldite medium. Ultrathin sections were cut using a Leica UC6 microtome, stained with lead citrate and observed under a ThermoFisher Scientific Talos L120C transmission electron microscopy operated at 120 kV. Digital acquisition was performed with a Ceta CMOS camera.

### Quantification of myelin and mitochondria

Morphological changes in myelin and mitochondria were assessed from confocal images with an ImageJ (version 1.54f) plugin “Mitochondria Analyzer”. Several measures, including counts, area, length, junctions, diameters, and endpoints, were examined according to a previous report [29]. The default block size for mitochondria was set to 1.25 μm and 50 μm for myelin sheath.

### Animal behavioural tests

#### Open field test

The Open Field test assesses the anxiety levels of mice. The platform is a large lidless box that is divided into two zones: center and periphery. In theory, anxious mice spend more time in the periphery (which provides greater protection), while curious ones explore the central region more. The test ran for 15 min for each mouse and individual movement was tracked via an overhead camera. Data such as ‘distance travelled’, ‘time in periphery/center’, and ‘distance in periphery/center’ were collected and analyzed.

#### Pole test

The pole test is a behavioural test that measures coordination and motor function in mice. A 50 cm pole with a stand was placed in the cage. Extra bedding was placed in the cage to protect the falling mice from the pole. The pole test was performed on 3 consecutive days. For the first 2 days, mice were placed on top of the pole and trained to walk down the pole. The training can be performed a few times until mice can walk down the pole by themselves. For the third day, the timings of each mouse walking down the pole for two rounds were recorded for analysis. 

#### Rotarod test

The Rotarod test measures coordination and motor skill learning. Mice were placed on a barrel-shaped platform that rotated slowly at first (4 rpm), which then gradually accelerated over 5 min (to 40 rpm). Each mouse had to balance for as long as possible before falling off. There is a pressure plate below the apparatus, which stops the time recording when the mouse has activated it. The time taken for each mouse to fall was measured here. The longer the duration, the better the coordination and motor skill learning.

#### Cylinder test

This cylinder test measures the vertical movement of mice. Each mouse was placed in an empty cylinder and a 5-minute timer was simultaneously started. When mice are placed in a novel environment, they tend to stand on their hind legs to better smell the surroundings (a behaviour known as rearing). In this test, the number of rears were recorded for each mouse.

### Cell culture

Human neuroblastoma cell line SH-SY5Y (ATCC^®^ CRL-2266™) was maintained in Dulbecco’s Modified Eagle Medium (DMEM) (Gibco) supplemented with 10% Fetal Bovine Serum (FBS) (Gibco) 1% L-GlutaMAX (Gibco), and 1% penicillin-streptomycin and incubated at 37^o^C with 5% CO_2_. Plasmid transfection in SH-SY5Y was conducted using Lipofectamine^®^ LTX transfection reagent (Invitrogen) following the manufacturer’s instruction. Transfected cells were used for immunoblotting or immunocytochemical staining assays 2 days post-transfection. Quantification of N2C protein aggregation was performed using the AggreCount plugin in ImageJ (version 1.54f) [30].

### Oxygen consumption rate (OCR) assay

The OCR assay was performed using Agilent Seahorse XF Cell Mito Stress Test Kit (#103015-100) following the manufacturer’s instructions. 10,000 SH-SY5Y cells were reverse transfected with pAAV-N2C-polyG plasmids using the Lipofectamine^®^ LTX transfection reagent and seeded in a 96-well Seahorse XF cell culture microplate. After overnight culturing in a 37^o^C incubator, the microplate was placed in an Agilent Seahorse XF Analyzer for analysis following the standard protocol. The assay data were automatically calculated by the analysis and the results were plotted using the GraphPad Prism 8.

### Immunohistochemistry (IHC) staining

The mouse brain specimens were fixed with 4% paraformaldehyde and then soaked in 30% sucrose before use. The fixed specimens were then sliced by a cryostat machine (Leica) and mounted on the slides. Before immunostaining, the slides with specimens were blocked (using 1% BSA in 1xPBS, 0.1% Triton X-100) for 30 minutes at room temperature after washing with 1x PBS. The specimens were then incubated with primary antibody against p62, (Cell Signaling, #39749), MBP (Cell Signaling, #83683), TH (Millipore, #AB9702), α-synuclein (BD Transduction, #610787), p-α-synuclein (S129) (Cell Signaling, #23706S), and Tuj1 (Millipore, #1637) at 4°C overnight. After washing 3 times with PBS, the slides were incubated with secondary antibody Goat anti-Rabbit IgG Alexa Fluor 488 (Thermo Fisher, #A11034), Goat anti-Chicken IgG Alexa Fluor 568 (ThermoFisher, #A11041), Donkey anti-Mouse IgG Alexa Fluor 555 antibody (Thermo Fisher, #A31570) and Goat anti-Rabbit IgG Alexa Fluor 647 (Thermo Fisher, #A21245) for 2 hours at room temperature in dark [31]. Nuclear counterstaining was performed with 4’, 6-diamidino-2-phenylindole dihydrochloride (DAPI) (Sigma-Aldrich, #B2261) or Hoechst 33,342 (Thermo-Fisher). Images were obtained using an FV3000 confocal microscope (Olympus).

### Immunocytochemistry staining

Cells were fixed with 4% paraformaldehyde for 15 minutes at room temperature. After washing with 1x PBS, cells were permeabilized in 1% BSA in 1x PBS with 0.1% Triton X-100 for 30 minutes. Samples were then incubated with respective primary antibodies in a humid chamber at 4^o^C overnight. After washing with 1x PBS for 3 times, the samples were incubated with secondary antibodies and 4’, 6-diamidino-2-phenylindole dihydrochloride (DAPI) (Sigma-Aldrich, #B2261) for 2 h at room temperature. Images were obtained using an Olympus FV3000 confocal microscope. Laser channels at wavelengths of 405 nm, 488 nm, 561 nm, and 640 nm were used in this study.

### Cell viability assay

Cell viability assay was performed using cell proliferation reagent WST-1 according to the manufacturer’s instruction (Roche, 11644807001). Briefly, 24 h after Lipofectamine^®^ LTX (Thermo Fisher, #15338100) transfection, 20,000 cells were seeded into a 96-well cell culture dish and incubated for another 24 h. 10 µl cell proliferation reagent WST-1 was added to each sample in the 96-well cell culture plate, and cells were incubated for 0.5 to 1 h at 37 °C in a 5% CO_2_ incubator. After 1 min of agitation on a shaker, the absorbance of samples was measured in a microplate reader at 450 nm wavelength.

### Plasmids construction

Full-length N2C2 (NCBI accession number NM_001364013.1), including N2C-30G, N2C-45G, N2C-63G27S, and N2C-90G, was synthesized by Cyagen Biosciences Inc. A common pair of primers 5’ CCCAAGCTTCTCCCCATGTGGATCTGCCCA 3’ and 5’ TGTGGATCCATTCTCATCGTGTTCTTTTCCATTCC 3’ was used to amplify the N2C-polyG and subcloned into the pAAV-uN2C-99G plasmid (a gift from Dr Charlet-Berguerand [13]) at the HindIII and BamHI sites. After Sanger sequencing, we found that only 13G was present in N2C-30G and 89G was present in 99G constructs. Thus, we finally obtained N2C-13G, N2C-45G, N2C-32G13S, N2C-89G, and N2C-63G27S.

### RNA extraction and Q-PCR

Total RNA from tissue or blood was isolated using QIAzol (#5346994, QIAGEN) according to the manufacturer’s instructions. Total RNA was reverse transcribed using an iScript cDNA Synthesis kit (#170–8891, BIO-RAD). Q-PCR was performed in a LightCycler system (Roche) with 2× All-in-One qPCR master mix (#QP001-01, GeneCopoeia) and specific primers. The expression of each gene was defined from the threshold cycle, and the relative expression levels were calculated using the 2^−△△Ct^ method. The following primer pairs were used: N2C1: 5’ ACCCCCGCGCATGCATTGCA 3’ and 5’ CCACACAAGTCCCACCATTCT 3’; N2C2: 5’ ACCCCCGCGCATGTGTCGAGA 3’ and 5’ CCACACAAGTCCCACCATTCT 3’.

### Western blot

Cell lysis and immunoblot were performed as previously described [32]. Twenty micrograms of each protein sample were loaded onto a sodium dodecyl sulfate-polyacrylamide gel electrophoresis (SDS-PAGE) gel for separation using a Bio-Rad Mini-PROTEAN^®^ System. To separate and show both low and high-molecular-weight proteins, three layers of gels (from top to bottom, 4-8%-12%) were used to cast each SDS-PAGE gel. Afterwards, proteins were transferred onto a 0.45 μm polyvinylidene difluoride (PVDF) membrane (Millipore, #IPVH00010) for immunoblotting. PVDF membranes were blocked with 5% skim milk in 1x Tris Buffered Saline with Tween 20 (TBST) for 1 h and incubated with primary antibodies overnight. The antibodies used in this study were tyrosine hydroxylase (TH) (Millipore, #MAB318), LRRK2 (Abcam, #ab133474), α-synuclein (BD Transduction Lab, #610787), p62 (Cell Signaling, #39749), DRP1 (Cell Signaling, #8570), LC3II (Abcam, #ab243506), CD11b (Cell Signaling, #49420S), MBP (Cell Signaling, #83683), MOG (Merck Millipore, MAB5680), β-actin (Santa Cruz, #AC-15). Blots were then incubated with 1:3000 diluted secondary antibodies in BSA for 2 h. Anti-mouse IgG HRP (GE Healthcare, #NA931V) and anti-rabbit IgG HRP (GE Healthcare, #NA934V) were used. Autoradiography was performed using Enhanced chemiluminescence (ECL) substrate and West Femto substrate (Thermo Scientific) and developed by a ChemiDoc MP imaging System (Bio-Rad). Signal intensity of immunoblots was quantified using ImageJ (NIH, Bethesda, MD).

### Dot blot

N2C-polyG overexpression vectors were transfected into SH-SY5Y cells by Lipofectamine LTX (Thermo Fisher, #15338100) in a 6-well cell culture plate. After 48-hour transfection, cells were collected and lysed in a Triton-soluble buffer (20 mM Tris, 150 mM NaCl, 1% Triton X-100, and protease and phosphatase inhibitor cocktails (MedChemExpress, #HY-K0010 and #HY-K0022). Lysates were centrifuged at 16,000 g for 10 min and supernatants were collected as soluble fractions. The precipitates were further dissolved in a buffer containing 8 M urea in PBS at 37^o^C for 30 min and ultrasonicated on ice. After being centrifuged at 16,000 g for 10 min, the supernatants were collected as insoluble fractions. The proteins were denatured at 37^o^C for 5 min and dropped onto a nitrocellulose membrane. The customized N2C peptide (GRCWRSGCAARPP) and Rabbit polyclonal antibody N2C targeting both N2C1 and N2C2 were generated by BiomatiK. After blocking in 5% non-fat milk, the membrane was incubated with the N2C2 antibody at 4^o^C overnight. After washing, the membrane was incubated with anti-rabbit IgG HRP (GE Healthcare, #NA934V) for 1 h. Autoradiography was performed using Enhanced chemiluminescence (ECL) substrate and West Femto substrate (Thermo Scientific) and developed by a ChemiDoc MP imaging System (Bio-Rad). Signal intensity of immunoblots was quantified using ImageJ (NIH, Bethesda, MD).

### Statistical analyses

For each experiment, at least three independent measurements were performed. All statistical analyses were performed using GraphPad Prism 8 software. Data were presented as mean ± SD for all statistical analyses except for the whole-cell patch clamp recording, which used mean ± SEM. For 2 group comparison, a two-way Student’s *t* test was used to compare the difference between the 2 groups. For multiple group comparisons under one experimental condition, one-way ANOVA with Tukey’s post *hoc* test was used to compare the differences between groups. For multiple group comparisons under two experimental conditions, two-way ANOVA with Tukey’s post *hoc* test was used. The statistical significance levels were set at *P < 0.05, **P < 0.01, and ***P < 0.001.

## Results

### N2C-polyG intermediate repeat with serine promotes protein aggregation and neuronal cell death in vitro

In the previous screenings of *NOTCH2NLC* GGC repeats in ET and PD patients [5, 7], we identified the presence of both isoform 1 (N2C1) and isoform 2 (N2C2) of *NOTCH2NLC* in our patients (Supplementary Fig. 1). To ascertain the ratio of N2C1 to N2C2 among patients with different repeat expansions, we extracted peripheral blood mononuclear cells (PBMCs) from patients carrying N2C GGC_90_ and GGC_108_ as well as a healthy individual carrying GGC_25_. These cells were then reprogrammed into iPSCs, and quantitative PCR (qPCR) analysis of patient iPSC-derived neurons was performed to determine the relative abundance of N2C1 and N2C2. The ratio of mRNA levels for N2C1 to N2C2 in N2C-25G, N2C-90G, and N2C-108G was 20.7:1, 11.8:1, and 17.6:1, which were equal to 4.6%, 7.8%, and 5.4% of N2C2 in these samples, respectively (Supplementary Fig. 2). Patients with high GGC repeats had relatively lower N2C1 levels compared to healthy individuals carrying GGC_25_ (Supplementary Fig. 2). The percentages of N2C2 in our patient iPSC-derived neurons are lower than previous reports with 10% and 16% of the N2C2 to total N2C in human cells [15, 18]. However, the role of N2C2 in neurodegeneration is unknown. Although N2C2 is less predominant in humans, its role of N2C2 in neurodegeneration is unknown and it may play an important role in the pathogenesis of NREDs.

**Fig. 1:**
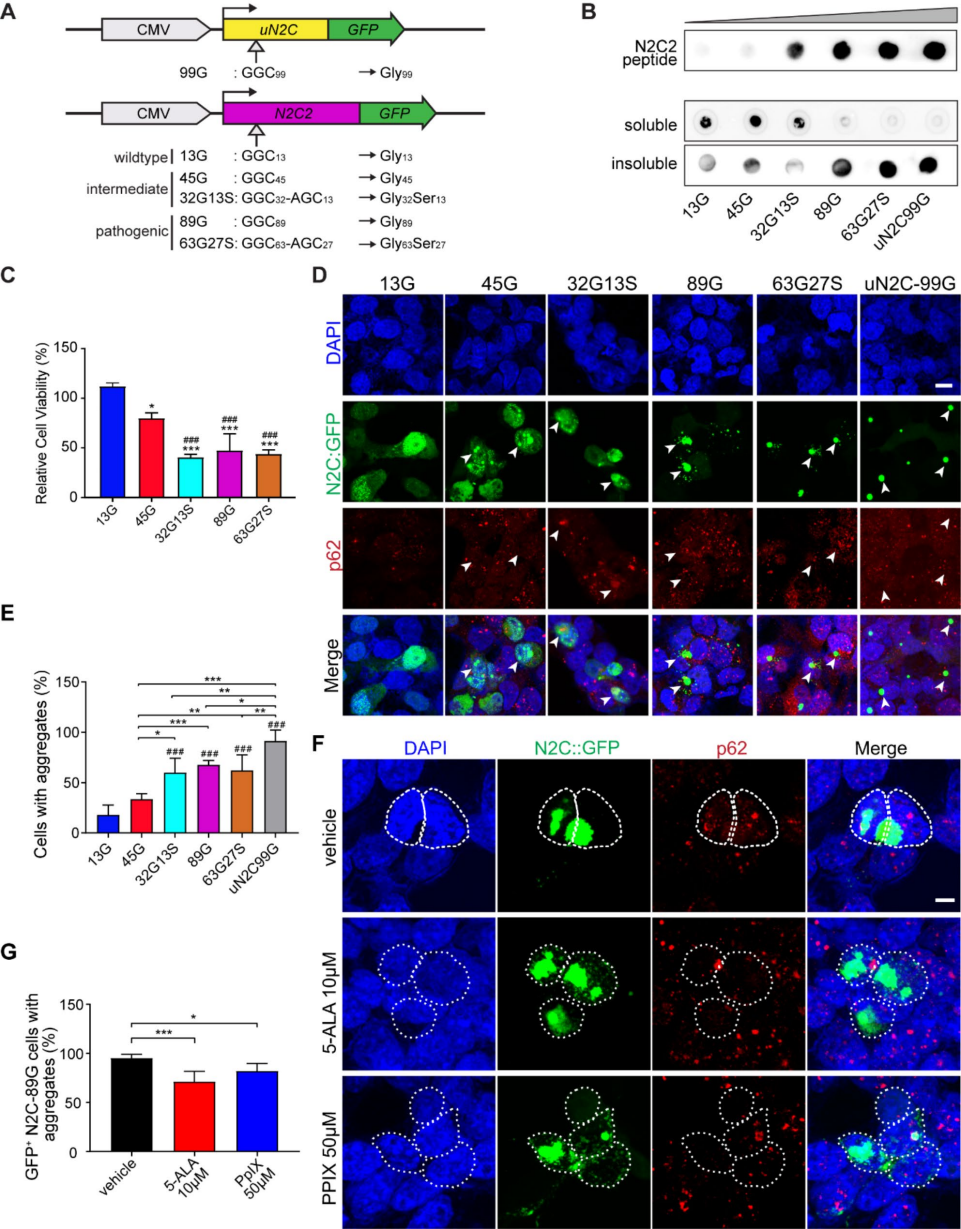
N2C-polyG intermediate repeat with serine insertion promotes protein aggregation and increases neuronal cell death in vitro. (**A**) Illustration of the N2C expression constructs used in vitro. (**B**) Dot-blot image of pure N2C peptide (GRCWRSGCAARPP) (0, 6.125, 12.5, 25, 50, and 100 ng) and urea-soluble and insoluble fractions of the lysates of Lipofectamine LTX transfected SH-SY5Y carrying N2C-13G, -45G, -32G13S, -89G, -63G27S, and uN2C-99G. A customized N2C antibody targeting both N2C1 and N2C2 proteins was used for the detection of the N2C-polyG proteins. (**C**) WST1 cell viability assay was performed after transfecting SH-SY5Y cells with N2C-polyG plasmids. * represents comparisons between each of the other groups and 13G. # represents the comparisons between other groups and 45G. (**D**) SH-SY5Y cells were transfected with N2C vectors carrying different expansion repeats. Immunocytochemical staining was performed with a p62 antibody. White arrowheads represent cells colocalized with N2C: GFP and p62. Scale bar = 20 μm. (**E**) Quantification of N2C-polyG protein aggregation shown in **D**. N2C-polyG protein aggregations were defined as green fluorescence dots between 1 and 5 μm. Cell numbers counted: 13G = 118, 45G = 147, 32G13S = 57, 89G = 101, 63G27S = 90, and uN2C-99G = 105. # represents comparisons between other groups and 13G. (**F**) Treatment of N2C-89G transfected SH-SY5Y cells for 30 h with 50 µM PPIX and 10 µM 5-ALA. Scale bar = 10 μm. (**G**) Quantification of protein aggregations shown in **F**. Cell numbers counted: vehicle = 268, 10 µM 5-ALA = 259, 50 µM PPIX = 128. Data are presented as the mean ± SD, with *n* = 3 per group. *P < 0.05, **P < 0.01, and ***P < 0.001 by one-way ANOVA with Tukey’s post *hoc* test

Patients with different GGC repeat expansion sizes and with AGC insertion display diverse disease presentations [5, 12], thus we posit that the GGC repeat size and the presence of AGC insertion may modulate neurotoxicity. In PD patients, the average frequency of AGC insertion in N2C GGC repeat expansions is 25% ± 12% [5]. To investigate the effect of the size of GGC repeats encoding polyglycine (polyG) in N2C2 and the average number of AGC insertions encoding serine (S) in neurodegeneration, we generated a series of N2C2 expression constructs carrying different sizes of GGC and AGC insertion. This includes N2C2-13G (the wildtype, normal GGC repeats), 45G and 32G13S (GGC intermediate repeat and with 28.8% serine, similar to the average percentage of AGC insertion within N2C GGC repeat expansion in PD patients), 89G and 63G27S (pathogenic GGC repeat expansion, and with 30% serine), as well as the uN2C-99G (high GGC repeat expansion, a gift from Dr. Charlet-Berguerand [13]) as a control for N2C1 (Fig. 1A). The N2C-polyG is tagged with an eGFP to its C-terminal as a fusion protein for easy visualization. We found that with the increase of repeat expansion size, the amount of soluble N2C protein decreased and the insoluble N2C increased, suggesting the formation of insoluble N2C-polyG aggregation in higher repeats (Fig. 1B). To examine the effect of N2C repeat expansion on neuronal cell death, as previously reported [13, 15], a WST1 cell viability assay was performed. Consistently, N2C-45G, -32G13S, -89G, and -63G27S showed more cell death compared to 13G (Fig. 1C, **28**.7%, 64.0%, 57.8%, and 61.0% respectively). 32G13S, 89G, and 63G27S showed further neuronal cell death compared to 45G (Fig. 1C, **35**.4%, 29.2%, and 32.3% respectively). Notably, 32G13S showed more cell death as compared to 45G, which indicates that serine insertion can accelerate the neurotoxicity in intermedium size repeat expansion. To investigate the effect of intermediate repeat with serine on N2C protein aggregation formation, we quantified protein aggregation in SH-SY5Y cells. Similar to uN2C-99G, higher repeat polyG forms intracellular inclusion (Fig. 1D). We found that from 45G onwards, the N2C starts to form protein aggregations, which are between 1 and 5 μm in diameter. With the increase of repeat expansion numbers, the percentages of cells carrying aggregates increased (Fig. 1D **and E**). 32G13S has a higher percentage of cells with aggregates compared to 45G (Fig. 1D **and E**, 77.5% increased). These results suggest that higher repeat expansion tends to form insoluble neurotoxic protein aggregates. Additionally, serine insertion promotes protein aggregation.

Collectively, our data indicate similar intracellular inclusion and neurotoxicity induced by N2C2-polyG as compared to N2C1-polyG. In addition, the results suggest a correlation between the size of repeat expansions and the total amount of aggregates formed by the N2C-polyG protein. Specifically, larger polyG repeats and the presence of serine insertions are associated with the increased formation of N2C-polyG protein aggregates. Notably, the increased size of aggregates, particularly those with larger polyG repeats, correlates with a heightened propensity to induce cellular death.

Fragile X-related tremor/ataxia syndrome (FXTAS) is another member of the polyG neurodegenerative disease family, caused by GGC repeat expansion in 5’-UTR of *FMR1* [33, 34]. Previous studies reported that 5-aminolevulinic acid (5-ALA) and its metabolite protoporphyrin IX (PpIX) can inhibit FMR-polyG protein aggregation in vitro and ameliorate learning and motor dysfunction in FMR-polyG mice [35]. It is of interest to know whether the two FXTAS drugs are effective in inhibiting N2C-polyG protein aggregation. To test this hypothesis, we treated N2C-89G transfected SH-SY5Y cells with 5-ALA and PpIX. Remarkably, we observed that treatment with 10 µM of 5-ALA and 50 µM of PpIX effectively mitigated intranuclear N2C protein aggregation after 24 h (Fig. 1F). The percentage of cells exhibiting N2C-89G protein aggregates significantly decreased compared to that of the control group (Fig. 1G, reductions of 24.23% and 12.78% for 10 µM 5-ALA and 50 µM PpIX, respectively). These findings indicate that 5-ALA and PpIX treatment mitigates N2C-polyG protein aggregates. This implies a shared pathogenic pathway and therapeutic approach for polyG diseases, including FMR-polyG and NREDs.

### N2C-polyG intermediate repeat with serine impairs mitochondrial respiration in vitro

**Fig. 2:**
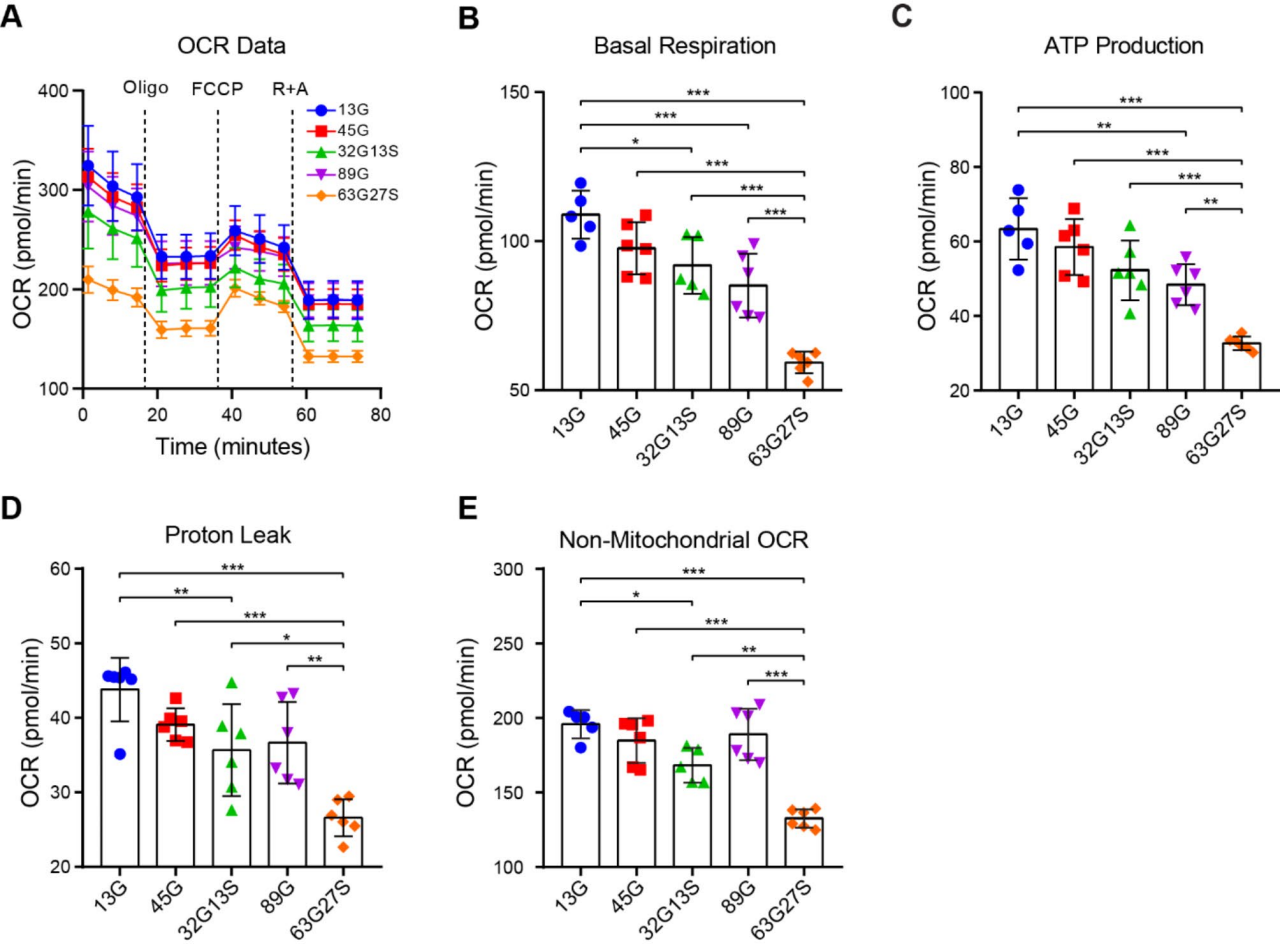
N2C-polyG intermediate repeat with serine insertion impairs mitochondrial respiration in vitro. SH-SY5Y cells transfected with N2C-13G, -45G, -32G13S, -89G, and − 63G27S were seeded in a 96-well Seahorse XF cell culture microplate and cultured for 24 h. The OCR assay was performed using the Agilent Seahorse XF Cell Mito Stress Test Kit. The overall OCR data (**A**), basal respiration (**B**), ATP production (**C**), proton leak (**D**), and non-mitochondrial OCR (**E**) are shown. Data are presented as the mean ± SD, with *n* = 6 per group. *P < 0.05, **P < 0.01, and ***P < 0.001 by one-way ANOVA with Tukey’s post *hoc* test

DNA damage repair activity was reported to be compromised in uN2C-polyG cells [13]. A recent study showed that uN2C-100G interacted with mitochondrial RNA binding protein LRPPRC and mitochondrial oxidative phosphorylation was downregulated in uN2C-100G transgenic flies and NIID patient muscle biopsies [19]. Another study reported mitochondrial dysfunction in cardiac myocytes in a *NOTCH2NLC-(GGC)*_*98*_ transgenic mouse model [22]. We speculated that the N2C intermediate repeat with serine insertion also impairs mitochondrial function. We thus measured cellular oxygen consumption rate (OCR), a key parameter of mitochondrial function, in N2C-GGC repeat expansion vectors transfected SH-SY5Y cells (Fig. 2A) [36]. Results showed that the basal respiration rates decreased as the increase in GGC repeat number (13G vs. 32G13S, reduction of 17.0%; 13G vs. 89G, reduction of 23.8%) and serine insertion further deteriorated the basal respiration (89G vs. 63G27S, reduction of 25.8%) (Fig. 2B). Intermediate 45G did not show a statistically significant difference in basal respiration, however, with the 13-serine insertion within 45 repeats, the basal respiration rate was significantly decreased in 32G13S compared to 13G (Fig. 2B, reduction of 17.0%). After oligomycin treatment, which blocks complex V and inhibits ATP synthesis, ATP production was significantly decreased with the increase of GGC repeat expansions (13G vs. 89G, reduction of 14.9%; 45G vs. 63G27S, reduction of 25.9%), which was further reduced by serine insertion (89G vs. 63G27S, reduction of 15.8%) (Fig. 2C). Similarly, with the increase of GGC repeat expansion, proton leak and non-mitochondrial OCR were both decreased, which were further reduced by serine insertion (Fig. 2D **and E**). Intermediate repeat with serine insertion showed a significant decrease in both proton leak and non-mitochondrial OCR compared to 13G.

Taken together, the OCR assay revealed a reduction in basal energetic demand, ATP production by mitochondria, and non-mitochondrial respiration in cells with elevated N2C-GGC repeat lengths. Additionally, the detrimental impact was further exacerbated by the presence of serine insertions. Notably, an intermediate repeat combined with serine insertion was observed to diminish OCR. These outcomes imply that both high GGC repeat expansions and intermediate repeat with serine in N2C detrimentally affect mitochondrial function, leading to a disruption in energy supply. This disruption is implicated in the observed cellular death and neurodegeneration within the spectrum of NREDs.

### N2C-polyG intermediate repeat with serine triggers protein aggregation and exhibits early PD-like phenotypes in mice

**Fig. 3:**
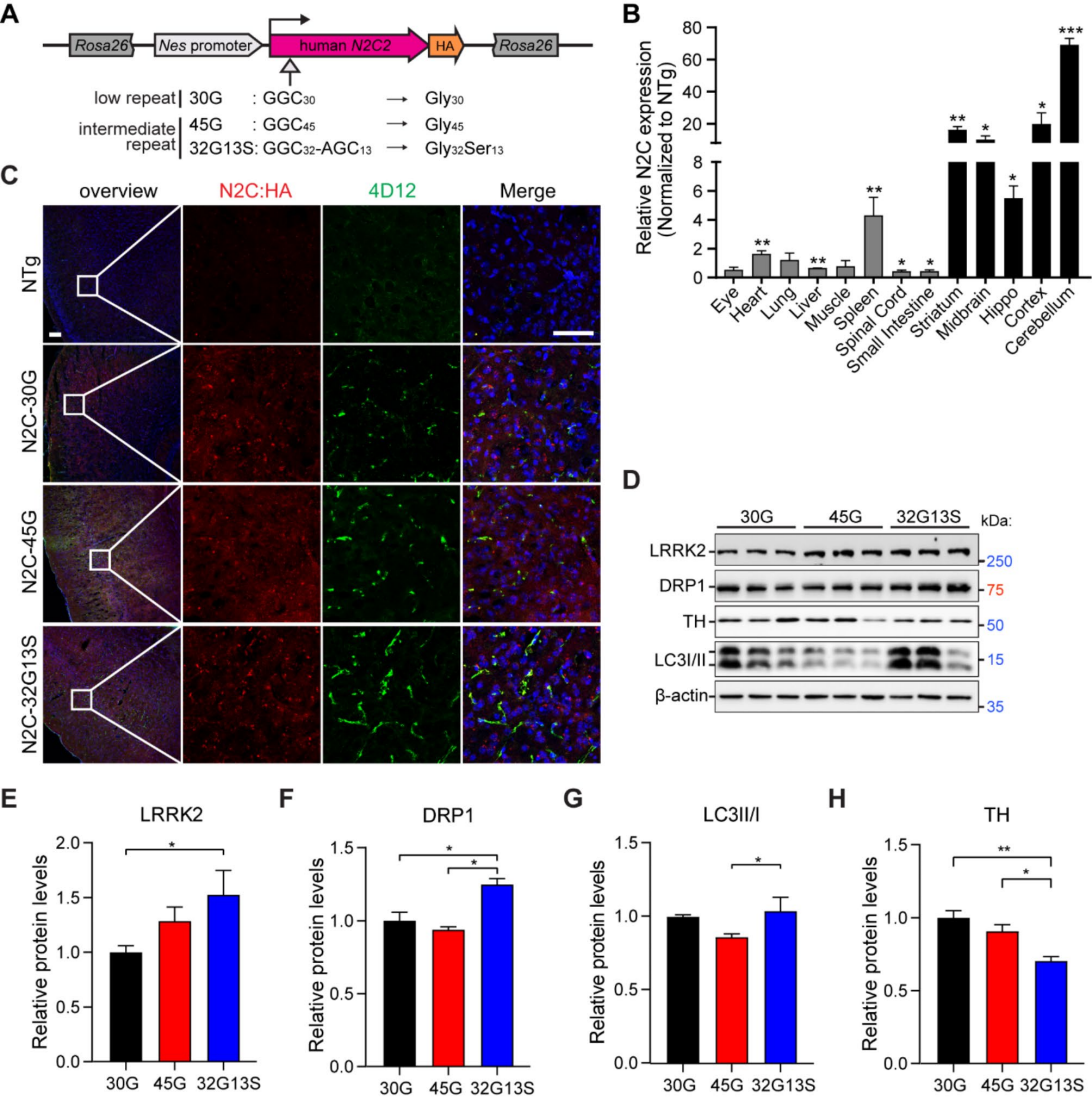
N2C-32 S triggers N2C-polyG protein aggregation and resembles PD pathogenesis in transgenic mice. (**A**) Illustration of the N2C-polyG transgenic mice. The human N2C2 coding sequence, carrying 30G, 45G, or 32G13S, is driven by the human *Nes* promoter and fused with an HA-tag. This sequence is inserted into the *Rosa26* locus of the C57BL/6 N mouse genome by CRISPR/Cas9-mediated genome editing. Heterozygous mice of both genders were used for experiments. (**B**) Tissues were collected from adult N2C-32G13S and non-transgenic (NTg) mice for qPCR analysis. Relative N2C expression levels in N2C-32G13S were normalized to NTg. Data are presented as the mean ± SD, with *n* = 3 per group. *P < 0.05, **P < 0.01, and ***P < 0.001 by two-tailed Student’s *t* test. (**C**) Cortical tissues from 8-month-old NTg, N2C-30G, -45G, and − 32G13S transgenic mice were used for immunostaining. Both HA (red) and 4D12 (green) antibodies were used to detect N2C-polyG protein. Scale bar = 100 μm. (**D**) Cortical tissues were used for immunoblotting. (**E-H**) Quantification of LRRK2 (**E**), DRP1 (**F**), LC3II to LC3I ratio (**G**), and TH (**H**) from the analysis in **D**. Data are presented as the mean ± SD; *n* = 3 per group. *P < 0.05 and **P < 0.01 by one-way ANOVA with Tukey’s post *hoc* test

To further investigate the function and pathological contribution of the N2C-polyG repeat expansion with intermediate repeat and AGC insertion, we generated a series of N2C-polyG transgenic mouse lines, including N2C-30G (low repeat), -45G (intermediate repeat), and − 32G13S (intermediate repeat with 28.9% of serine insertion) (Fig. 3A). To create the transgenic mouse models, a *Nes*-driven human *N2C2*-expressing cassette was inserted into the *ROSA26* locus in C57BL/6 N mice by CRISPR/Cas9-mediated genome engineering (Fig. 3A). The human *Nestin* promoter allows the expression of the protein in the central nervous system (CNS) [24]. The qPCR results showed that the N2C-polyG proteins were highly expressed in the CNS of adult N2C-polyG mice (Fig. 3B). Immunostaining showed that N2C protein expression was detected by both HA and N2C antibody 4D12 (a gift from Dr. Charlet-Berguerand [13]) in the cortical region of N2C transgenic mice, but not in non-transgenic (NTg) mice (Fig. 3C). Importantly, we found that 45G formed small aggregates as indicated by the 4D12 antibody. The 32G13S mice showed larger fiber-like aggregates across cells as compared to both N2C-30G and 45G (Fig. 3C). This result suggests that the N2C-polyG with serine insertion promotes the formation of fiber-like aggregation in the cortex of 32G13S mice, which implies a hitherto unknown yet possibly important role of the serine insertion in the neurodegeneration and pathophysiology in NREDs.

To investigate the pathogenic pathways induced by N2C-polyG, we conducted immunoblotting analysis using cortex tissues from 12-month-old N2C transgenic mice. Since NREDs exhibit parkinsonism syndromes and NIID has mitochondrial dysfunction, we thus examined several related protein markers, including LRRK2, one of the most common genetic causes of PD, DRP1, and LC3, that are critical for autophagy and mitochondria function, as well as tyrosine hydroxylase (TH), the rate-limiting enzyme for the conversion of amino acid tyrosine to dopamine [32, 37, 38]. Results showed that LRRK2 protein levels were increased in 32G13S mice compared to 30G (Fig. 3D **and E**, 52.5% increase). 32G13S mice had elevated protein levels of DRP1 and LC3 (II to I) compared to 45G mice (Fig. 3D, F, **and G**, 33.1% increase of DRP1 and 20.7% increase of LC3). We also observed a reduction in TH level in 32G13S compared to both 30G and 45G (Fig. 3D **and H**, 29.7% and 22.5% reductions, respectively). These results are consistent with previous observations of mitochondrial dysfunction in vitro and clinical features of parkinsonism, dementia, and neuronal loss in PD and other NREDs.

**Fig. 4:**
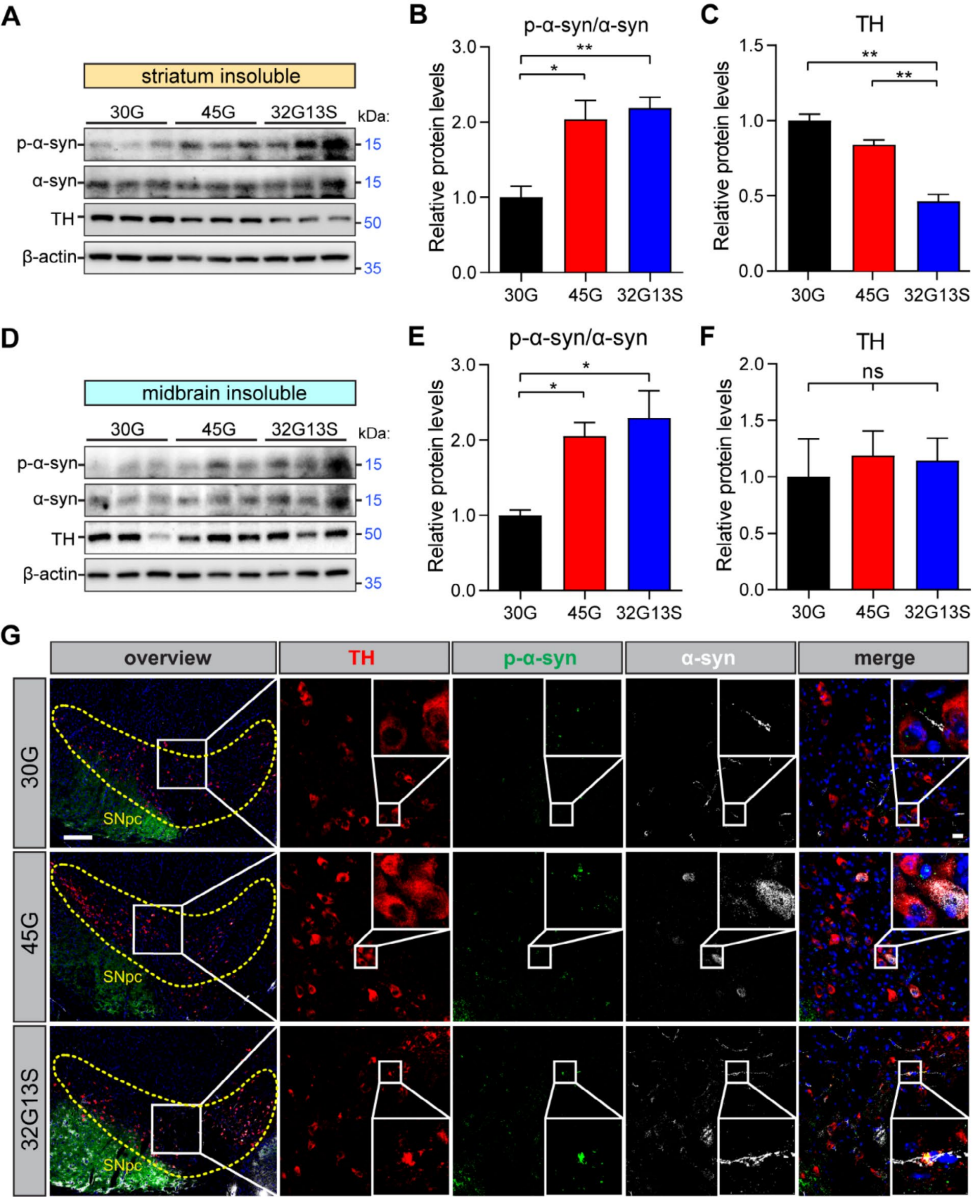
N2C-polyG intermediate repeat with serine displays early PD-like phenotypes. **(A)** Western blot analysis of insoluble proteins from the striatum of 12-month-old N2C-30G, -45G, -32G13S mice. (**B-C**) Quantification of p-α-synuclein (S129) to α-synuclein (**B**) and TH (**C**) in **A**. **(D)** Western blot analysis of insoluble proteins from the midbrain of 12-month-old N2C-30G, -45G, -32G13S mice. (**E-F**) Quantification of p-α-synuclein (S129) to α-synuclein (**E**) and TH (**F**) in **D**. (**G**) IHC staining of 12-month-old N2C-30G, -45G, -32G13S mouse midbrain region by TH (red), p-α-synuclein (S129) (green), and α-synuclein (grey). The yellow dotted outline represents the substantia nigra pars compacta (SNpc) region. The scale bar in the left overview panel is 200 μm. The scale bar in the zoomed image is 20 μm. Data are presented as the mean ± SD; *n* = 3 per group. Ns = no significance. *P < 0.05 and **P < 0.01 by one-way ANOVA with Tukey’s post *hoc* test

Given that 32G13S mice showed elevated LRRK2, impaired mitochondrial activity, and reduced TH level, consistent with typical parkinsonism phenotypes, we next ought to examine the midbrain region of the mice, where the formation of α-synuclein deposition and the loss of dopaminergic (DA) neurons occur in PD [32, 37]. Immunoblotting analysis showed increased phosphor-α-synuclein Ser129 levels in both 45G and 32G13S mice compared to 30G in both soluble and insoluble fractions of the striatum and midbrain tissues (Fig. 4A, B, D, E, and Supplementary Fig. 3). Interestingly, TH reduction was only observed in the cortex (Fig. 3H) and striatum but not midbrain in 32G13S mice (Fig. 4A, C, D, F, and Supplementary Fig. 3). Furthermore, IHC staining revealed large fiber-like α-synuclein protein aggregates in the midbrain of 32G13S but not 45G mice (Fig. 4G), without changes in TH levels. This further confirmed immunoblotting results. No significant change in p62 and DRP1 protein levels was observed in both striatum and midbrain among N2C transgenic mice (Supplementary Fig. 4). These results indicate that DA neuronal loss mainly occurs in the cortex and striatum but not midbrain, which differs from the report in patients with high *NOTCH2NLC* GGC repeats [6]. This may be due to impaired dopaminergic projections and compensatory mechanisms. It was reported that axonal impairment in the striatum appears before the visible loss of neurons in the substantia nigra in transgenic PD rats expressing mutated forms of α-synuclein [39]. These results suggest that N2C intermediate repeat with serine induces early PD-like pathogenesis in mice.

Together, the CNS-specific expression of N2C intermediate repeat with serine insertion accelerates protein aggregation, induces mitochondrial dysfunction-associated neurotoxicity, and exhibits early Parkinson-like characteristics in mice. This aligns with our clinical observation that PD patients carrying N2C-polyG intermediate repeat with serine usually present with more severe disease symptoms than non-carrier PD patients [5].

### N2C-polyG intermediate repeat with serine triggers neuronal hyperexcitability and impairs motor function in mice

**Fig. 5:**
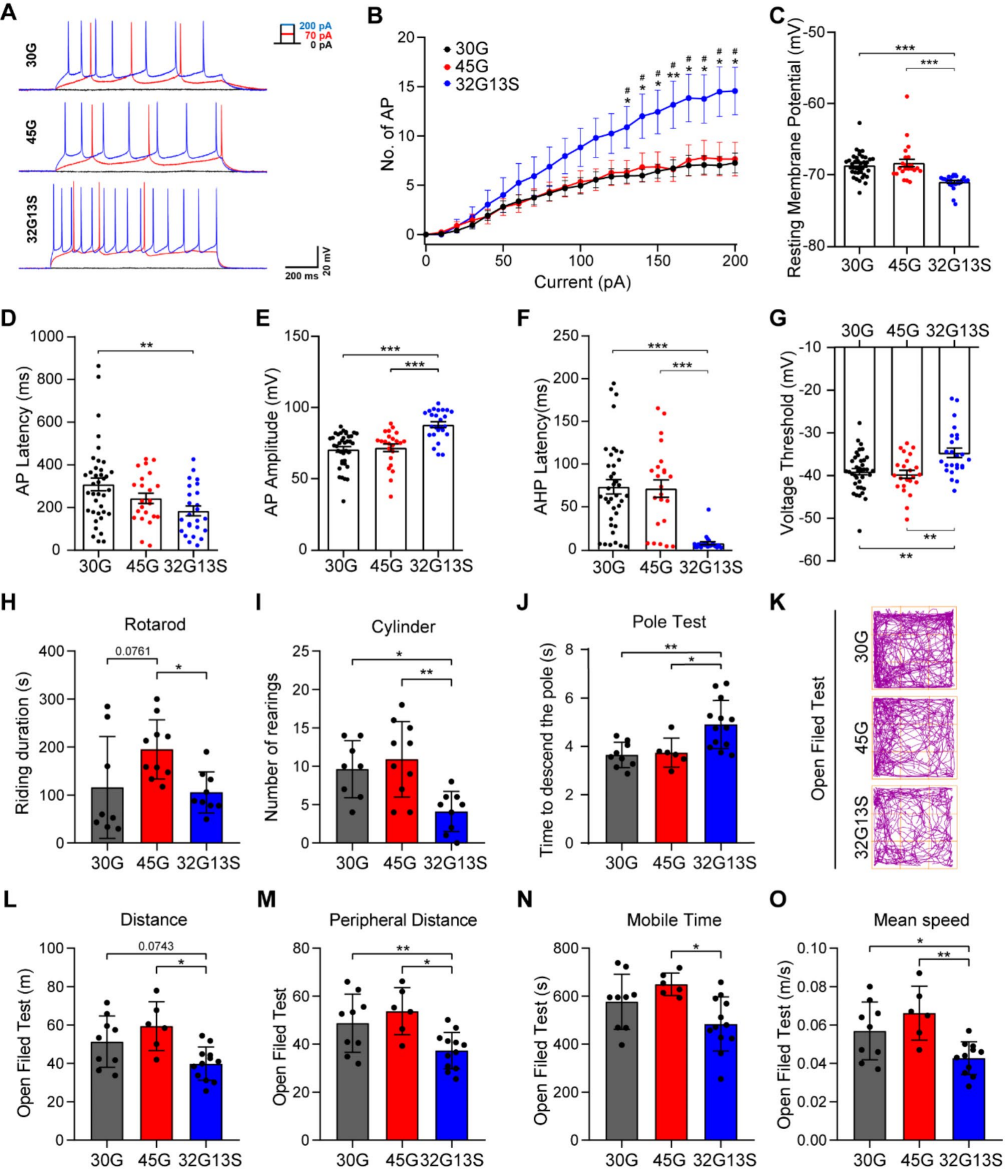
N2C-polyG intermediate with serine insertion induces neuronal hyperexcitability and motor deficits in mice. (**A-G**) Prefrontal cortex cells from 16-month-old N2C-30G, -45G, or -32G13S transgenic mice were used for electrophysiological tests. (**A**) Representative voltage response obtained from the medial prefrontal cortex (mPFC) of mouse brains. (**B**) Number of action potentials (AP) at different voltages. * represents comparisons between 32G13S and 30G. # represents comparisons between 32G13S and 45G. (**C-G**) Basic intrinsic neuronal parameters: resting membrane potential (mV) (**C**), AP latency (**D**), AP amplitude (**E**), after hyperpolarisation (AHP) latency (**F**), and AP voltage threshold (**G**) are significantly different in 32G13S PFC compared to N2C-30G and N2C-45G neurons. No differences were observed in input resistance (Rin), AP duration, or AHP amplitude among N2C-30G, N2C-45G, or N2C-32G13S (Supplementary Fig. 5A-C). N2C-30G, *n* = 38; N2C-45G, *n* = 23; N2C-32G13S, *n* = 25. Three independent mice per genotype were used for data collection. One-way ANOVA with Tukey’s post *hoc* test was used for statistical analysis. Data are presented as the mean ± SEM. * *P* < 0.05, ** *P* < 0.01, and *** *P* < 0.001. (**H-O**) Behavioural tests were performed in 12-month-old N2C-30G, -45G, and − 32G13S mice in Rotarod (**H**), Cylinder (**I**), Pole Test (**J**), and Open Field Test (**K**-**O**). (**K**) Representative walking trails of mice in the Open Field Test. Total distance (**L**), peripheral distance (**M**), Mobile time (**N**), and Mean speed (**O**) travelled during the Open Field Test are shown. N2C-30G, *n* = 9; N2C-45G, *n* = 6; N2C-32G13S, *n* = 13. Data are presented as the mean ± SD. *P < 0.05 and **P < 0.01 by one-way ANOVA with Tukey’s post *hoc* test

Previous studies have reported locomotor deficits and motor function loss in mouse and fly models carrying uN2C-polyG or via AAV infection [13, 19, 20]. In high repeat mice, these deficits correlate with impaired temporal control in motor behaviour [40]. The activity of the medial prefrontal cortex (PFC), which plays a vital role in the control of goal-directed and decision-dependent actions [41], is significantly disrupted by the loss of dopamine signaling with the progression of PD and clinical motor defects [42]. Firstly, we investigated the impact of intermediate N2C repeat with serine on cortical function by performing whole-cell patch clamp recordings of the prelimbic and infralimbic areas of the medial PFC in the 16-month-old N2C mice (Fig. 5A). We observed an increase in excitability of the 32G13S neurons compared to the 30G and 45G neurons in the PFC at high current inputs (beyond 130 pA) (Fig. 5B). This is likely the outcome of the decreased resting membrane potential (Fig. 5C), action potential (AP) latency (Fig. 5D), AP amplitude (Fig. 5E), and reduced afterhyperpolarisation (AHP) latency (Fig. 5F), which allows an easier induction of AP, despite comparable basal ionic conductance (Rin input resistance), AP duration, and AHP latency, which affects AP duration (Supplementary Fig. 5A-C). Furthermore, the enhancement in voltage threshold (Fig. 5G) may contribute to the delayed enhancement in the excitability of the 32G13S neurons, which is above the current stimulus of 130 pA (Fig. 5B). Next, we divided the PFC neurons into the prelimbic layer (PL) and infralimbic layer (IL) neurons and found that the hyperexcitability of the PFC neurons is mainly contributed by IL neurons (Supplementary Fig. 5D and E). The IL cortex has connections to subcortical regions that influence the autonomic nervous system. Changes in IL electrophysiological activity might disrupt the balance between cortical motor planning and autonomic regulation, which can impair fine motor control [43]. The hyperexcitability observed in 32G13S is probably a functional compensation within the motor system to compensate for deficient activation of the striato–mesial–frontal projections, due to the gradual loss of PFC neurons with the protein aggregation [44].

To investigate the impact of N2C-polyG intermediate repeat with serine on animal behaviour, behavioural tests were conducted on 12-month-old N2C-polyG mice. Results showed that 32G13S mice had shorter riding times in the rotarod test (Fig. 5H, **45**.9% less) and fewer rearings in the cylinder test (Fig. 5I, **62**.3% less) than the 45G mice. 32G13 mice also took longer time to descend the pole than both 30G and 45G mice in the pole test (Fig. 5J, **34**.5% and 31.2%, respectively). In the open field test, 32G13S mice walked shorter total and peripheral distances (22.4% and 30.6%, respectively) and exhibited less mobile time and mean speed than 45G mice (25.4% and 35.5%) (Fig. 5K **to O**). These results suggest that N2C-polyG intermediate repeat with serine insertions induces motor deficits in 32G13S mice. Taken together, our data indicate that intermediate repeat with serine triggers hyperexcited action potentials in the cortex of 32G13S mice and induces locomotor deficits in mice.

### Mitochondrial-related pathways and myelin sheath components are dysregulated in the cortex of N2C-32G13S mice

**Fig. 6:**
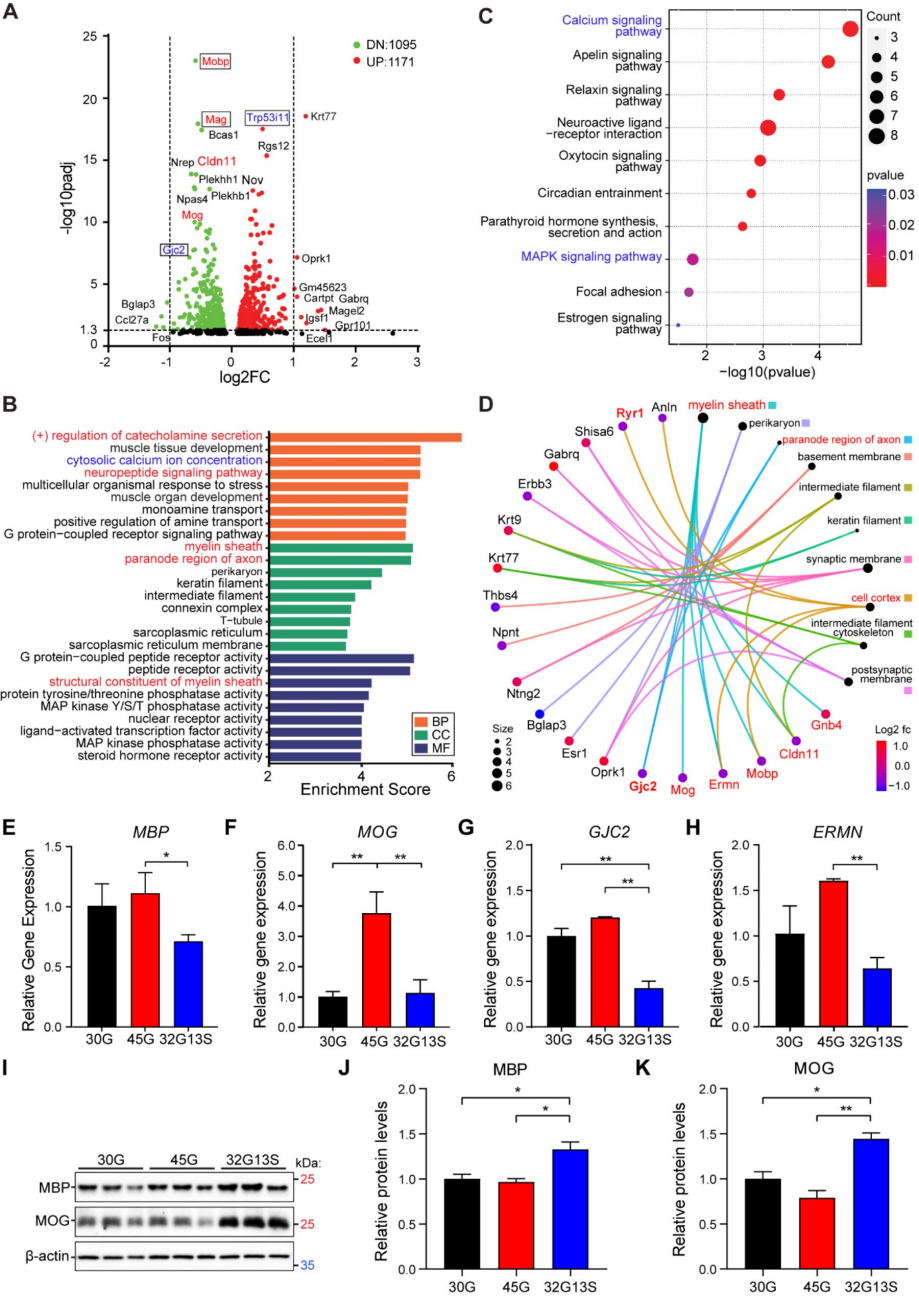
Mitochondrial-related pathways and myelin sheath component genes are dysregulated in N2C-32G13S mice. Cortical tissues from 12-month-old N2C transgenic mice were used for bulk RNA sequencing. *n* = 3 mice per group. (**A**) Volcano plot showing differentially expressed genes between N2C-32G13S and N2C-45G mice at padj < 0.05 and fold change < 0.67 or > 1.5. Top dysregulated genes are highlighted, with those related to mitochondrial function shown in blue. (**B**) For differentially expressed genes between N2C-32G13S and N2C-45G mice, 54 top dysregulated genes are shown in biological process (BP), cellular component (CC), and molecular function (MF) by gene ontology (GO) term analysis. (**C**) Top dysregulated KEGG pathways between N2C-32G13S and N2C-45G mice. (**D**) Connections between top differentially expressed genes and cellular components. Genes highlighted in red are myelin sheath components. (**E-H**) Quantitative PCR was performed for myelin sheath component genes *MBP* (**E**), *MOG* (**F**), *GJC2* (**G**), and *ERMN* (**H**) using cortical tissues from 12-month-old N2C mice. (**I**) Cortical tissues were used for immunoblotting. (**J-K**) Quantification of MBP (**J**) and MOG (**K**) from western blot in **I**. Data are presented as the mean ± SD, *n* = 3. *P < 0.05 and **P < 0.01 by one-way ANOVA with Tukey’s post *hoc* test

Having demonstrated N2C-polyG aggregation and motor deficits in N2C mice, we next aimed to elucidate potential pathogenesis-associated pathways in these mice. Following behavioural testing, some 12-month-old N2C-polyG mice were sacrificed, and cortical tissue was collected for bulk RNA sequencing. To focus on disease-associated pathways, we specifically analysed differentially dysregulated genes in the 32G13S and 45G mouse groups (Supplementary Fig. 6). A total of 2,266 genes (1,171 upregulated and 1,095 downregulated) were identified as differentially expressed in 32G13S vs. 45G mice (Fig. 6A). Among these top differentially expressed genes, we found several mitochondrial function-related genes, including the Gap Junction Protein Gamma 2 (*Gjc2*) and Tumor Protein p53 Inducible Protein 11 (*Trp53i11*). Gjc2 encodes Connexin 47, a protein predominantly expressed in oligodendrocytes, which are involved in myelination in the CNS. Connexins, including Gjc2, are known to influence mitochondrial calcium homeostasis and intercellular communication [45]. As one of the p53 inducible proteins, Trp53i11 is involved in the regulation of mitochondria-related processes, particularly through its role in apoptosis and cellular stress responses [46]. Notably, dysregulation was observed in biological processes, including cytosolic calcium ion concentration, which is important for mitochondrial function, cellular components (myelin sheath and paranodal region of axons), and molecular functions (structural constituents of the myelin sheath) in 32G13S compared to 45G mice (Fig. 6B). Among the top KEGG pathways, the calcium signaling pathway and MAPK signaling pathway are closely related to mitochondrial function (Fig. 6C). Calcium plays a crucial role in regulating mitochondrial function, including ATP production, oxidative stress, metabolism, and apoptosis [47, 48]. The MAPK signaling pathway is a critical regulator of mitochondrial function by modulating apoptosis, mitochondrial biogenesis, and oxidative stress [49]. Moreover, several commonly dysregulated genes among these processes were identified, including *MOG*,* GJC2*, and *ERMN* (Fig. 6D). Furthermore, the bulk RNA transcriptomic analysis revealed that these myelin sheath component genes are downregulated in N2C-32G13S mice (Supplementary Table 2). Taken together, our transcriptomic profile suggests mitochondrial dysfunction and myelin sheath dysregulation in the cortex of 32G13S mice.

Mitochondria play a crucial role in maintaining the health and functionality of myelinated axons, and disturbances in mitochondrial function can contribute to the degeneration of both neurons and the myelin sheath [50]. To validate myelin dysregulation in the cortex of 32G13S mice, qPCR analysis was conducted to measure the mRNA levels of these genes. The results demonstrated a significant decrease in myelin sheath component genes *MBP*, *MOG*, *GJC2*, and *ERMN* in 32G13S compared to 45G mice at the mRNA level (Fig. 6E-H). Notably, these myelin sheath component genes were also downregulated in patient iPSC-derived neurons (Supplementary Fig. 7A-D). Immunoblotting analyses were further conducted to examine protein levels. Surprisingly, results revealed significantly enhanced protein levels of both MBP (37.4% increase) and MOG (82.5% increase) in the 32G13S cortex compared to 45G (Fig. 6I-K). Both MBP and MOG protein levels also appeared to be higher in patient iPSC-derived neurons (Supplementary Fig. 7E). It has been shown that MBP mRNA is decreased in mouse demyelination models [51, 52]. We speculate that this observation could be the result of a compensatory mechanism to maintain crucial neuron function and signal transmission by the production of myelin sheath in response to mitochondrial impairment-induced neuronal loss in the cortex.

### N2C-polyG intermediate repeat with serine induces mitochondrial dysfunction-associated hypermyelination in mice

**Fig. 7:**
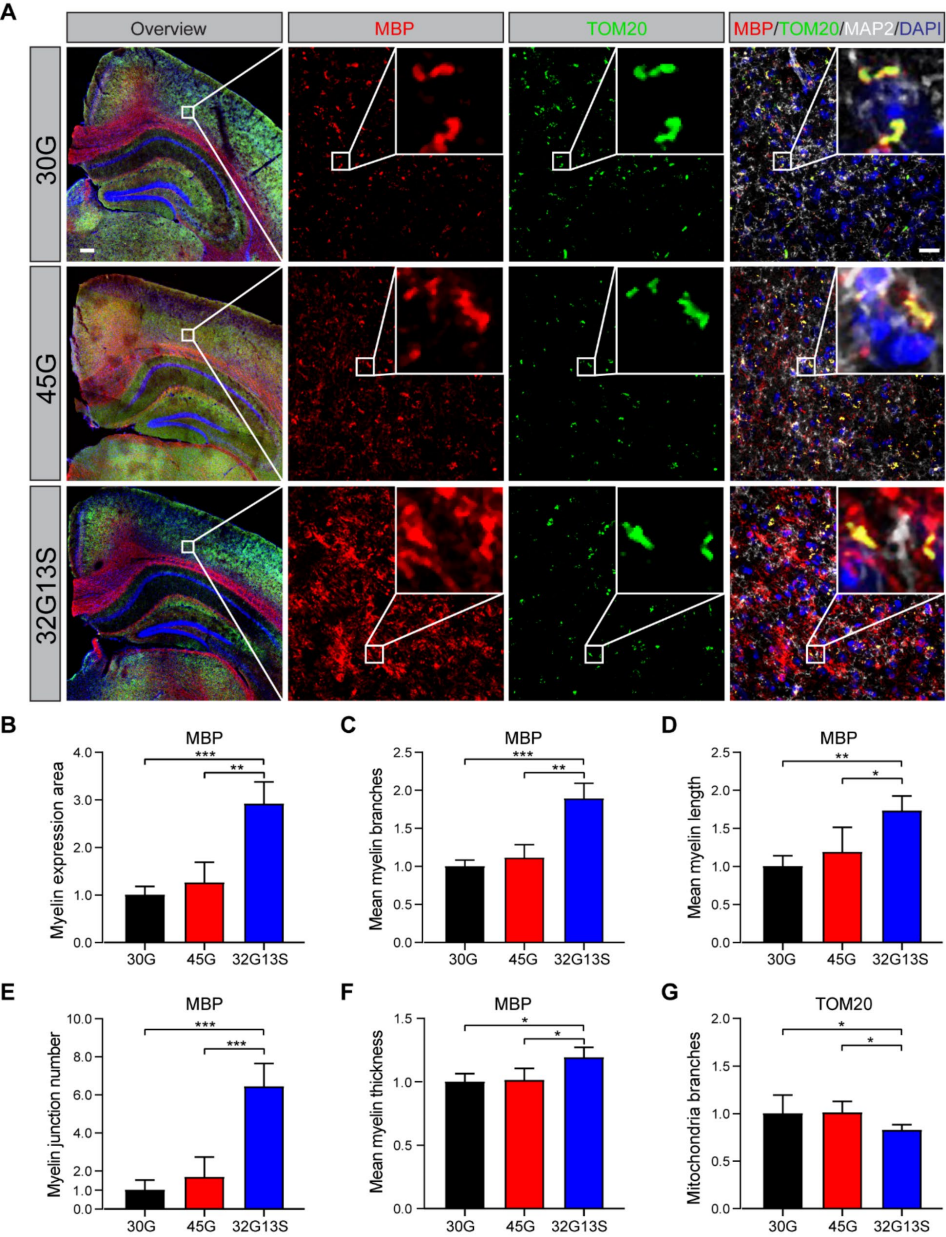
N2C-polyG intermediate repeat with serine induces mitochondrial dysfunction-associated hypermyelination in mice. (**A**) IHC staining of 12-month-old mouse brains. Immunofluorescence was detected using MBP (red), Tom20 (green), and Map2 (grey) antibodies. Scale bar = 200 μm in the overview image. Scale bar = 20 μm in the zoomed image. Quantification of myelin and mitochondria was conducted using an ImageJ plugin macro “Mitochondria Analyzer”. Cells quantified: 30G *n* = 4078, 45G *n* = 3949, 32G13S *n* = 4848. MBP and Tom20 signals represent myelin and mitochondria, respectively. (**B-G**) MBP expression area (**B**), myelin branches (**C**), myelin length (**D**), myelin junction number (**E**), mean myelin thickness (**F**), and mitochondrial branches (**G**) were measured and quantified by the Mitochondria Analyzer. Data are presented as the mean ± SD, *P < 0.05, **P < 0.01, and ***P < 0.001 by one-way ANOVA with Tukey’s post *hoc* test

Myelin is a lipid-rich material enveloping nerve cell axons, facilitating signal transduction and action potential propagation. Myelin Basic Protein (MBP) is the second most abundant myelin protein in the CNS, constituting about 30% of the dry protein mass in CNS myelin. Myelin sheath injury was reported to occur prominently in both motor and sensory nerves of NIID patients [53, 54]. Disruptions in the myelination network have been observed in the cingulate cortex of PD cases [55]. However, with ageing in neurodegenerative diseases, myelin disruption occurs through the formation of out-folding myelinosomes or hypermyelination [56]. Our study revealed mitochondrial dysfunction and myelin sheath component protein upregulation in the cortex of N2C-32G13S mice (Figs. 3D and 6I). High metabolic activity in myelinating cells necessitates increased mitochondrial ATP production [57]. Mitochondrial function is impaired in both myelinating neurons and oligodendrocyte precursor cells [58]. To further confirm the upregulation of myelin sheath protein components, we performed IHC staining of the 12-month-old N2C mice and found that MBP protein level was significantly increased in the cortex of 32G13S mice compared to both 30G and 45G mice (Fig. 7A **and B**, 26.0% and 190% more, respectively), which is consistent with the immunoblotting results (Fig. 6I **and J**). These results suggest hypermyelination in the cortex of 32G13S mice. To further investigate the correlation between myelin upregulation and mitochondrial dysfunction, we co-stained the mouse brain sections using MBP and Tom20 (a mitochondrial outer membrane protein). We found that the signals for MBP and TOM20 overlap with MAP2, a mature neuron marker, highlighting the presence of these proteins in the cortical neuron layer. In the cortical region, Tom20 is colocalized with MBP (Fig. 7A). In addition, 32G13S mice exhibited more myelin branches (Fig. 7C), longer myelin lengths (Fig. 7D), more myelin junctions (Fig. 7E), and thicker myelin sheaths (Fig. 7F) than both 30G and 45G mice in the cortical region, suggesting hypermyelination or over-ensheathment of axons in the 32G13S cortex. Meanwhile, 32G13S mice displayed fewer mitochondrial branches, indicative of an impaired mitochondrial network (Fig. 7G), which is in alignment with Western blot results showing reduced mitochondrial ATP production in vitro (Fig. 2) and impaired mitochondrial fission in vivo (Fig. 3). Together, all these data demonstrates that hypermyelination is associated with mitochondrial dysfunction in the 32G13S mouse cortex.

**Fig. 8:**
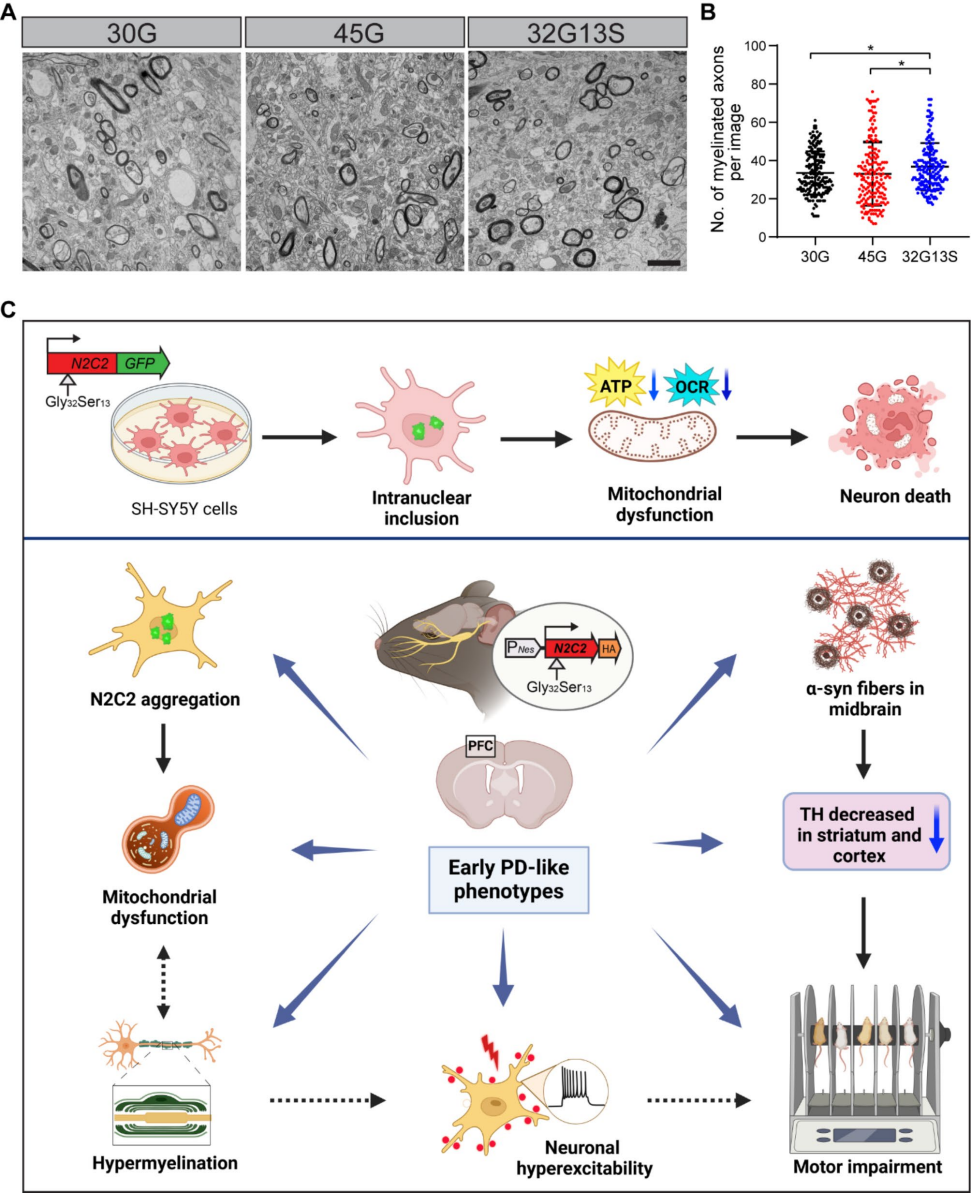
N2C-polyG intermediate repeat with serine induces hypermyelination in mice. Mouse cortical tissues near the corpus callosum were used for transmission electron microscopy (TEM). (**A**) Representative images of 30G, 45G, and 32G13S mouse cortex are shown at a magnification of 4300×. The scale bar is 2 μm. (**B**) Quantification of the number of myelinated axons under TEM. Myelinated axons are identified as the axons with dark circles in the images shown in **A**. Three mice were used for each group. Each dot represents one TEM image, as shown in **A**. Total image numbers: 30G = 187, 45G = 181, 32G13S = 199. Total myelinated axons counted: 30G = 5894, 45G = 5572, 32G13S = 6272. Data are presented as the mean ± SD, *P < 0.05 by one-way ANOVA with Tukey’s post *hoc* test. (**C**) **A graphical summary illustrates that N2C-polyG intermediate repeat with serine induces neurotoxicity and early PD-like neurodegeneration in vitro** **and in vivo**. Overexpression of the N2C-polyG intermediate repeat with serine promotes the formation of intranuclear inclusions, leading to mitochondrial dysfunction and subsequent neuronal cell death in vitro. In N2C-32G13S transgenic mice, this intermediate N2C-polyG with serine triggers N2C protein aggregation and early PD-like pathophysiological changes. These include α-synuclein fiber-like aggregates in the midbrain, loss of TH neurons in the striatum and cortex, and neuronal hyperexcitability with mitochondrial dysfunction-related hypermyelination in the cortex, all contributing to motor impairment. Our findings offer novel pathophysiological insights into the clinical relevance of N2C-polyG intermediate repeats observed in early PD patients

To directly observe myelination status in the 32G13S cortex, we conducted transmission electron microscopy (TEM) to directly visualize axon myelination. We observed increased numbers of myelinated axons in the cortical region of the 32G13S mice (Fig. 8A **and B**), which is consistent with our immunoblotting (Fig. 6) and IHC staining results (Fig. 7) of increased myelination in 32G13S mice. We also detected enhanced MBP protein levels in the striatum but not the midbrain and decreased CD11b levels in both the striatum and midbrain in 32G13S mice, suggesting hypermyelination and decreased microgliosis (Supplementary Fig. 8). The hypermyelination could be the result of a compensatory mechanism in response to reduced ATP production induced by mitochondrial impairment. It was reported that hypermyelination precedes demyelination in demyelination mouse models [59, 60]. Demyelination was also reported in the latest high repeat N2C-polyG AAV injection mouse model [21]. This aligns with our hypothesis that N2C-polyG intermediate repeat with serine induces early PD-like phenotypes, while high N2C-polyG may promote more severe neurodegeneration. Altogether, our results suggest that N2C-polyG intermediate repeat with serine induces mitochondrial dysfunction-associated hypermyelination in mice. All these pathophysiological changes observed manifest an early stage of neurodegeneration in 32G13S mice, where unknown compensatory mechanisms, such as increased myelination and neuronal activity, cannot fully counterbalance mitochondrial impairment and microglial dysfunction.

## Discussion

N2C GGC repeat expansions are linked to an expanding spectrum of NREDs, including PD. The distinctive pathological feature of N2C-polyG expansion involves the presence of eosinophilic intranuclear inclusions within neurons and glial cells [5, 7]. Notably, patients diagnosed with ET (0.9%) [7] and PD (1.3%) [5] have been identified with pathogenic (> 60 repeats) and intermediate (40–60 repeats) N2C-GGC repeat expansions [6]. Additionally, AGC insertion was observed within the GGC repeat expansions, and the frequency of AGC insertion repeats within the GGC expansions differs between PD and NIID (25% vs. 8%) [5, 7]. This study investigates the pathogenic role of N2C-polyG intermediate repeat with serine insertion in neurodegeneration. We generated N2C transgenic mouse models with different configurations and showed that the N2C-polyG intermediate repeat with serine 32G13S accelerates neurodegeneration in vitro and in vivo compared to 45G alone. These effects include mitochondrial dysfunction, cell death, neuronal hyperexcitability, hypermyelination, and locomotor behavioural deficits in mice (Fig. 8C). These findings suggest that both repeat number and serine insertion within the expansion play an important role in neurodegeneration and may manifest clinically as early parkinsonism.

Pathogenic N2C GGC expansions in NREDs typically involve repeat expansion sizes exceeding 60 [1–3, 61–63]. Interestingly, the size of GGC repeat expansion units appears to correlate with different disease presentations in various NREDs. Patients with more than 200 GGC repeat expansions tend to exhibit a phenotype dominated by muscle weakness, while those with 100–200 GGC repeats predominantly display dementia or essential tremor, and patients with less than 100 GGC repeats are more likely to present with parkinsonism [3, 8, 64]. In this study, we demonstrated that an intermediate repeat expansion with 45 repeats, containing 27.8% serine insertion, induced early PD-like phenotypes,  mitochondrial dysfunction, cell death, neuronal hyperexcitability, and motor deficits in mice. These findings support our previous observations that PD patients carrying N2C GGC repeats with AGC insertion are associated with more severe disease presentations [5]. Our data also indicate that N2C starts forming cellular aggregations at 45 repeat units, consistent with the observations by Zhong et al. [15]. In N2C mouse models, N2C-32G13S mice exhibited a trend of motor behavioural deficits at 8 months old, which became statistically significant at 12 months old, highlighting the significant role of intermediate repeat with serine insertion in disease progression.

Our previous studies identified the presence of AGC insertion within N2C GGC repeat in essential tremor and PD [5, 7]. Here, we showed that, compared to the same size in both intermediate (45G) and high repeat expansions (89G), the presence of 28.9–30% AGC insertion led to more significant neurodegeneration, including cell death, mitochondrial dysfunction, and motor deficits. These results suggest a crucial role of AGC insertion in the pathogenesis and disease progression of NREDs. Indeed, the insertion of short nucleotide repeat expansions has been reported to be important in other short nucleotide repeat expansion-associated diseases. Most nucleotide interruptions tend to play a protective role to reduce the neurotoxicity induced by the short nucleotide repeat expansion such as Fragile X syndrome (FXS) [65], Huntington’s disease (HD) [66], and Spinocerebellar ataxias (SCAs) [67–69]. In FXS, the loss of an AGG interruption is a key mutational event that contributes to the formation of unstable alleles, increasing the risk of developing Fragile X syndrome [65]. In HD, the loss of the CAA interruption within the CAG repeat expansion is linked to a significantly earlier age of onset even when polyglutamine (polyQ) length is the same as in individuals with CAA interruptions [66]. Silent CAA or missense CAT insertion, both encoding histidine, in polyQ-encoding CAG repeat expansion have been shown to modulate the neurotoxicity of the CAG repeat expansion and delay the onset age in SCA1 [67]. In SCA2, CAA interruptions are crucial for stabilizing the SCA2 repeat region; their absence increases the likelihood of alleles becoming unstable and undergoing pathological expansion [68, 69]. These demonstrate the essential protective role of interruptions in repeat expansion disorders. On the other hand, interruptions within short repeat expansions could also have detrimental effects on neurotoxicity. CCGCGG insertion in CATCAG repeat expansion is highly penetrant and correlated with age of onset in SCA8 patients with family history [70]. The SCA8 CCGCGG insertion encoding arginine in the polyGln, increases polyAla and polySer protein levels and polyGln toxicity [70]. This is similar to our finding that AGC interruptions in N2C GGC intermediate repeat expansion augmented neurotoxicity of N2C-polyG, which heightened disease penetrance and displayed an early-PD like phenotype. One reason for the instability of the insertion relative to pure repeat expansion may be the involvement of DNA repair factors [71]. DNA repair factors are potential genetic modifiers in HD and other CAG repeat expansion diseases [72, 73]. Specific DNA repair factors regulate the degree of instability and severity of the disease [74, 75]. Interestingly, two DNA repair factors, Ku70 and Ku80, have been identified to directly interact with uN2C-polyG, although they are not fully responsible for the pathogenesis [13]. To investigate the molecular impact of AGC insertion in the N2C GGC repeat on the pathogenesis of NREDs, three-dimensional studies of the mRNA and protein structures of the novel N2C-polyG, both with and without the AGC insertion, will be necessary.

Compared to other N2C-polyG genetic animal models, our N2C GGC intermediate repeat mice have relatively milder neurotoxicity, including behavioural deficits and mortality. In Boivin et al.’s model, mice infected with AAV carrying constitutively expressed N2C-99G have severe motor deficits and die within 6 months post-infection [13]. Similarly, in the N2C-polyG transgenic *Drosophila* model, the survival rate decreases in UAS-driven high repeat N2C-polyG flies [19]. In addition, in a recent N2C-polyG mouse model, accompanied by motor deficits, N2C-98G mice died within 70 days after birth [20]. In our N2C mouse model, 32G13S mice showed motor deficits without a significant increase in mortality even at 18 months old. There are two possible reasons for the differences in our mouse models. First, compared to the high repeat expansions (> 98G) in these animal models, our mouse models contain only low to intermediate repeats (45G). We have demonstrated a positive correlation between repeat expansion size and neurotoxicity, which is consistent with the previous report [15]. It is well-known that the size of repeat expansions contributes to the pathogenesis and disease severity of other short nucleotide repeat expansion diseases [71]. Second, the N2C expression regions are different between the models. In all previous studies, high repeat N2C-polyG is constitutively and non-specifically expressed throughout the entire organism. However, in our study, the intermediate N2C repeat expansions are under the control of the human *Nes* promoter, which is specifically expressed in the CNS (Fig. 3) [24, 25]. NREDs affect multiple systems and display multi-system-associated clinical manifestations, including dementia, parkinsonism, muscle weakness, essential tremor, retinopathy, urinary dysfunction, and digestive dysfunction [9, 12]. Among these clinical features, neurodegenerative symptoms are the predominant phenotypes [11, 12, 64]. Systemic expression of N2C-polyG mimics the systematic effects of N2C-polyG; however, it complicates and interferes with the mechanistic study of its effects in the CNS. To rule out the impact of N2C-polyG outside of CNS, we specifically expressed N2C-polyG in the CNS, which may cause a relatively mild phenotype compared to ubiquitous expression. A longer lifespan also enables longitudinal studies on the effects of N2C-polyG on ageing and disease progression. Indeed, we observed a gradual progression of motor deficits in 8- to 12-month-old mice. The CNS-specific expression of intermediate N2C with AGC insertion (32G13S) exhibited parkinsonism phenotypes, including motor behavioural deficits, correlating with our clinical observation that N2C intermediate causes more severe disease conditions in PD patients. This aligns with the recent discovery that high GGC expansion mice exhibit tremors in behavioural studies [21].

In 32G13S mice, we found that in the midbrain, α-synuclein formed fiber-like protein aggregation, with no TH^+^ neuronal loss or mitochondrial damage observed. In the striatum, α-synuclein aggregation was detected together with TH^+^ neuronal loss, but without mitochondrial impairment. In the cortex, we detected TH^+^ neuronal loss, mitochondrial impairment, and N2C protein aggregation (Supplementary Table 3). In contrast to the traditional pathophysiological changes in PD models [6], our findings suggest that N2C intermediate repeat with serine induces early PD-like phenotypes in mice, which exhibit relatively milder pathophysiological presentations. Several studies have reported early PD-like phenotypes in mouse models, typically involving both motor and non-motor symptoms that emerge before significant neurodegeneration in the substantia nigra (SN) [39, 76, 77]. MPTP-treated mice exhibit early PD-like phenotypes, including dopaminergic dysfunction in the striatum and cortex, sometimes even in the absence of significant dopaminergic neuron loss in the substantia nigra​ [76]. In addition, it was reported that axonal impairment in the striatum appears before the visible loss of neurons in the SN in transgenic PD rats expressing mutated forms of α-synuclein [39].

One possible explanation could be the impairment of dopaminergic projections. If TH levels are unchanged in the midbrain, it implies that the dopaminergic neurons are still present and producing TH. However, the reduction in TH in the striatum and cortex suggests that dopaminergic projections to these regions are impaired. Several studies suggest that the earliest subcellular structures affected by α-synuclein pathology in diseased neurons may be their neurites and synapses. Specifically, axonal dysfunction and impaired axonal transport are thought to play a critical role in triggering synucleinopathies in PD. These disruptions in axonal pathways could be key contributors to the onset of the disease [76]. Another possibility could be the activation of compensatory mechanisms [78]. In the early stages of neurodegenerative processes, compensatory mechanisms in the midbrain might maintain TH expression to compensate for the loss of dopaminergic neurons in the cortex. However, this compensation may not extend to terminal regions (such as the striatum and cortex), where deficits in dopamine transmission become more evident. This compensatory increase in dopamine can help delay the onset of motor symptoms, even when there is cortical degeneration [76]. The hyperexcitability we observed in the prefrontal cortical neurons may be another compensatory mechanism triggered by neuronal loss in the cortex. Similar to our finding, in the *PARK7*^−/−^ PD mouse model, D2 receptor expression is significantly decreased in the prefrontal cortex, where hyper-neuronal activity is detected [79]. Overall, our findings suggest that early PD-like pathophysiological changes were triggered by intermediate repeats with serine in mice, which may be due to impaired dopaminergic projections and compensatory mechanisms.

Another interesting finding is that myelin sheath component genes are dysregulated in the cortex of 32G13S mice. In the CNS, the myelin sheath is a protective layer that coats the axons. Myelin is generated by oligodendrocytes and functions in electrical signal transduction between neurons, which is crucial for CNS function [80, 81]. Myelin loss is associated with α-synuclein accumulation in oligodendrocytes in PD [82]. In addition, myelination networks, including myelin genes and oligodendrocyte development, are found to be disrupted in the PD cingulate cortex, where myelinating oligodendrocytes are the main cell type [55]. It was reported that the primary injury site in cases of NIID patients, based on electrophysiological tests, is the myelin sheath in both motor and sensory nerves [53, 54]. Myelination network disruptions were also observed in the cingulate cortex of PD cases [55]. Interestingly, we found downregulated mRNA levels of myelin-related genes alongside upregulation of myelin in this study (Figs. 6 and 7). The discrepancy between decreased mRNA levels for MBP may be due to post-transcriptional regulatory mechanisms, such as changes in translation efficiency or translational control, which can result in higher protein levels even when mRNA levels are low. The increased protein levels of MBP and MOG could reflect a compensatory response to repair or regenerate myelin despite the decreased mRNA levels. This might occur if the cells are actively involved in myelin repair or turnover processes [83]. On the other hand, we also observed impaired mitochondrial function together with increased myelination in the cortex. Mitochondrial dysfunction can hinder cellular energy production and impact overall cellular processes, including those involved in myelin maintenance. Cellular stress or mitochondrial damage can trigger compensatory responses that affect myelin-related proteins [84]. The increase in MBP and MOG could be a stress response aimed at maintaining myelin integrity or protecting against stress-induced damage, even in the context of mitochondrial dysfunction.

Overall, we observed mitochondrial impairment, hypermyelination, neuronal hyperexcitability, and neuronal loss in the cortex. Mitochondria play a critical role in neuronal energy metabolism, calcium homeostasis, and the regulation of apoptotic pathways. Impaired mitochondrial function is associated with decreased ATP production, leading to energy deficits in neurons, which are highly energy-dependent [85]. In 32G13S mice, mitochondrial dysfunction induced by N2C intermediate repeat protein aggregation can impair cellular energy production and impact overall cellular processes, including those involved in myelin maintenance. Cellular stress or mitochondrial damage can trigger compensatory responses including neuronal hyperactivity (Fig. 5) and the production of myelin sheath-related proteins, including MBP and MOG (Figs. 6 and 7) in the cortex. Firstly, when mitochondria are impaired, neurons are less able to manage the energy demands of synaptic activity and membrane potential maintenance. This imbalance can lead to the overactivation of excitatory neurotransmission, resulting in neuronal hyperexcitability [86]. Secondly, mitochondria are essential for oligodendrocyte function, which is important for myelination; any dysfunction in mitochondrial metabolism can lead to abnormal myelination patterns.

Studies have suggested that impaired mitochondrial energy production can stimulate aberrant myelination as oligodendrocytes attempt to compensate for neuronal energy deficits [87]. Hypermyelination can disrupt the normal balance of synaptic signaling, leading to alterations in neuronal communication. This alteration of synaptic balance can result in increased neuronal excitability due to a reduced threshold for neuronal firing [88]. Notably, studies have shown that disruption of myelin occurs first through hypermyelination before demyelination, a more severe disease phenotype in neurodegeneration [56, 59, 60]. Moreover, hypermyelination and over-ensheathment of axons are caused by feedback control during myelinogenesis in cases of merosin deficiency [89].

Nonetheless, the mechanism on how N2C protein aggregation-induced mitochondrial dysfunction regulates myelination and neuronal activity remains unknown and requires further investigation. We speculate that N2C-polyG aggregates in the cortex impair mitochondria, leading to reduced ATP production and neuronal loss. In response to these damages in the cortex, cortical neurons attempt to compensate for the neuronal deficits through hyperexcitability and ensheathment of the axons, causing over-ensheathment or hypermyelination [90]. In turn, this compensation may impair dopaminergic projections from the midbrain to the cortex at the cortical terminal. Thus, we observed an early PD-like phenotype, with more severe pathophysiological changes in the cortex compared to the midbrain.

## Conclusions

In conclusion, we provide the first evidence that N2C-polyG intermediate repeat with serine promotes neurotoxicity in vitro and induces early PD-like pathophysiological changes in vivo. Moreover, intermediate N2C-polyG with serine induces mitochondrial dysfunction, hyperexcitability, hypermyelination, and motor deficits in transgenic mice. Our findings provide novel pathophysiological insights into the clinical implications of N2C-polyG intermediate repeat observed in early PD patients.

## Electronic supplementary material

Below is the link to the electronic supplementary material.

Supplementary Material 1

## Data Availability

The data that support this study are available from the corresponding authors upon request. N2C-polyG transgenic mice generated in this study will be made available freely for academic research purposes and may require a Materials Transfer Agreement. Source data are provided in this paper.

## Abbreviations

AAV: Adeno-associated virus
ET: Essential tremor
FTD: Frontotemporal disorder
HD: Huntington’s disease
IHC: Immunohistochemistry
iPSC: induced pluripotent stem cell
N2C: *NOTCH2NLC*
N2C1: *NOTCH2NLC* isoform 1
N2C2: *NOTCH2NLC* isoform 2
NIID: Neuronal intranuclear inclusion disease
NREDs: NOTCH2NLC-related repeat expansion disorders
OCR: Oxygen Consumption Rate
PBMC: Peripheral blood mononuclear cell
PD: Parkinson’s disease
PFC: Prefrontal cortex
polyG: Poly glycine
SCA: Spinocerebellar ataxia
SNpc: Substantia nigra pars compacta
uN2C: Upstream open reading frame of < Emphasis Type="Italic”> *NOTCH2NLC*</Emphasis>
