# Supplementary material for: NOTCH2NLC GGC intermediate repeat with serine induces hypermyelination and early Parkinson’s disease-like phenotypes in mice
Source: Mol Neurodegener. 2024 Nov 28;19:91. doi: 10.1186/s13024-024-00780-2 (PMC11603791; doi:10.1186/s13024-024-00780-2)
Supplement: Supplementary file 1 — Supplementary Material 1 [file 13024_2024_780_MOESM1_ESM.docx]

Supplementary Materials for

***NOTCH2NLC* GGC intermediate repeat with serine induces hypermyelination and early Parkinson's disease-like phenotypes in mice**

Haitao TU^1^, Xin Yi YEO^2^, Zhi-Wei ZHANG^1^, Wei ZHOU^3^, Jayne Yi TAN^4^, Li CHI^1,5^, Sook-Yoong CHIA^1^, Zhihong LI^1^, Aik Yong SIM^6^, Brijesh Kumar SINGH^7^, Dongrui MA^8^, Zhidong ZHOU^3,9^, Isabelle BONNE ^6,10,11^, Shuo-Chien Ling^9,12^, Adeline S.L. NG^4,9^, Sangyong JUNG^13^, Eng-King TAN^3,4,9,*^, Li ZENG^1,9,14, *^

^1^Neural Stem Cell Research Lab, Research Department, National Neuroscience Institute, 308433, Singapore

^2^Department of Psychological Medicine, Yong Loo Lin School of Medicine, National University of Singapore, 119228, Singapore

^3^Research Department, National Neuroscience Institute, Singapore General Hospital (SGH) Campus, 169856, Singapore

^4^Department of Neurology, National Neuroscience Institute, 308433, Singapore

^5^Hospital of Stomatology, Guanghua School of Stomatology, Guangdong Provincial Key Laboratory of Stomatology, Institute of Stomatology, Sun Yat-Sen University, Guangzhou Guangdong, 510080, China

^6^Electron Microscopy Unit, Yong Loo Lin School of Medicine, National University of Singapore, 117549, Singapore

^7^Laboratory of Hormonal Regulation, Cardiovascular and Metabolic Disorders, Duke-NUS Medical School, 169857, Singapore

^8^Department of Neurology, Singapore General Hospital, 169609, Singapore

^9^Neuroscience & Behavioural Disorders Program, DUKE-NUS Graduate Medical School, 169857, Singapore

^10^Department of Microbiology and Immunology, Yong Loo Lin School of Medicine, National University of Singapore, 117545, Singapore.

^11^Immunology Translational Research Programme, Life Sciences Institute, National

University of Singapore, 117456, Singapore.

^12^Department of Physiology, Yong Loo Lin School of Medicine, National University of Singapore 119077, Singapore

^13^Department of Medical Science, College of Medicine, CHA University, Seongnam, 13488, Republic of Korea

^14^Centre for Molecular Neuropathology, Lee Kong Chian School of Medicine, Nanyang Technology University, Novena Campus, 308232, Singapore

*Corresponding to: Li ZENG,

Neural Stem Cell Research Lab, Research Department, National Neuroscience Institute, 308433, Singapore

Email: [Li_Zeng@nni.com.sg](mailto:Li_Zeng@nni.com.sg);

Correspondence may also be addressed to: Eng-King TAN

Research Department, National Neuroscience Institute, Singapore General Hospital (SGH) Campus, 169856, Singapore

Email: [tan.eng.king@singhealth.com.sg](mailto:tan.eng.king@singhealth.com.sg)

**This file includes:**

**Supplementary Figure 1-8**

**Supplementary Table 1-3**

**Supplementary Figures**

**Supplementary Figure 1**


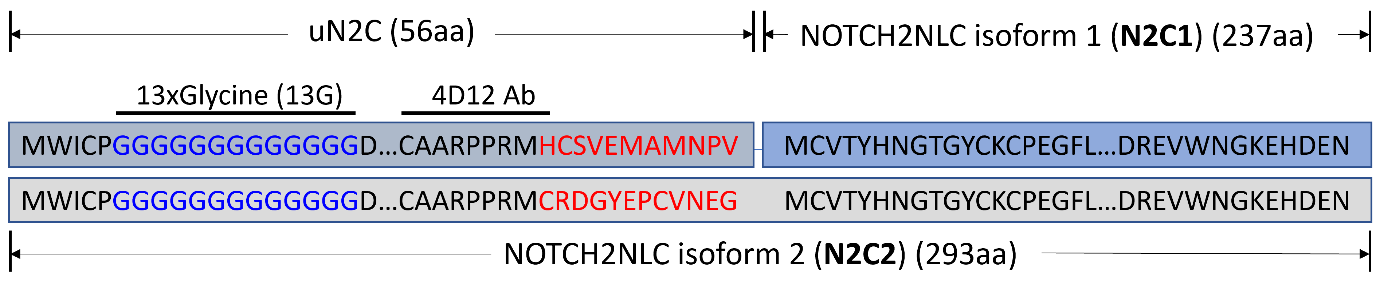


**Supplementary Fig. 1 *NOTCH2NLC* isoform 1 and 2 protein alignment.** Schematic diagram of two transcript variants of the human *NOTCH2NLC* gene. *NOTCH2NLC* isoform 1 (N2C1) protein has 237 amino acids (aa). *NOTCH2NLC* isoform 2 (N2C2) protein has 293 aa. The pathogenic protein uN2C (56 aa) is located within the 5’-UTR region of N2C1. Amino acids highlighted in blue represent the GGC repeat expansion. Amino acids in red represent the different amino acids in uN2C plus N2C1 compared to N2C2.

**Supplementary Figure 2**


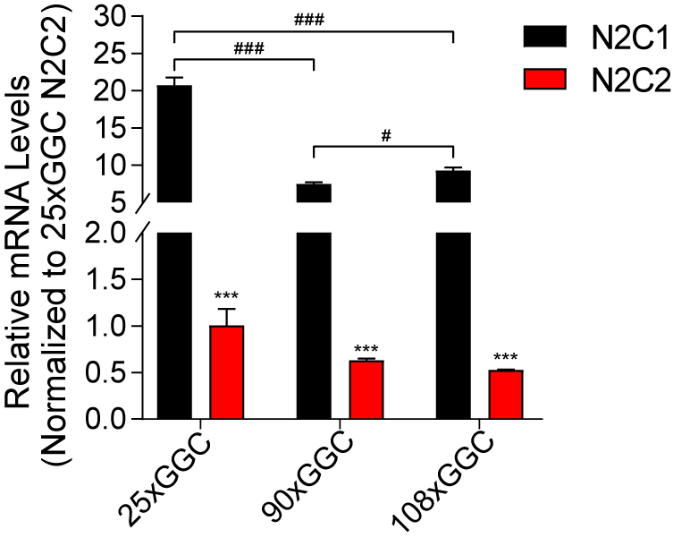


**Supplementary Fig. 2 qPCR analysis of the ratio of N2C1 and N2C2 in patient iPSC-derived neurons**. IPSC-derived neuron cDNAs extracted and converted from patients carrying 90 GGC and 108 GGC, as well as healthy individuals carrying 25 GGC were used for quantitative PCR analysis. N2C1 specific primer set (5’ ACCCCCGCGCATGCATTGCA 3’ and 5’ CCACACAAGTCCCACCATTCT 3’) and N2C2 specific primer set (5’ ACCCCCGCGCATGTGTCGAGA 3’ and 5’ CCACACAAGTCCCACCATTCT 3’) were used for quantitative PCR. Data are presented as the mean ± SD, n = 3. **P* < 0.05 and ****P* < 0.001 by two-way ANOVA with Tukey’s post *hoc* test. * represents comparisons between N2C1 and N2C2 within the same sample. # represents comparisons of N2C1 between different samples.

**Supplementary Figure 3**


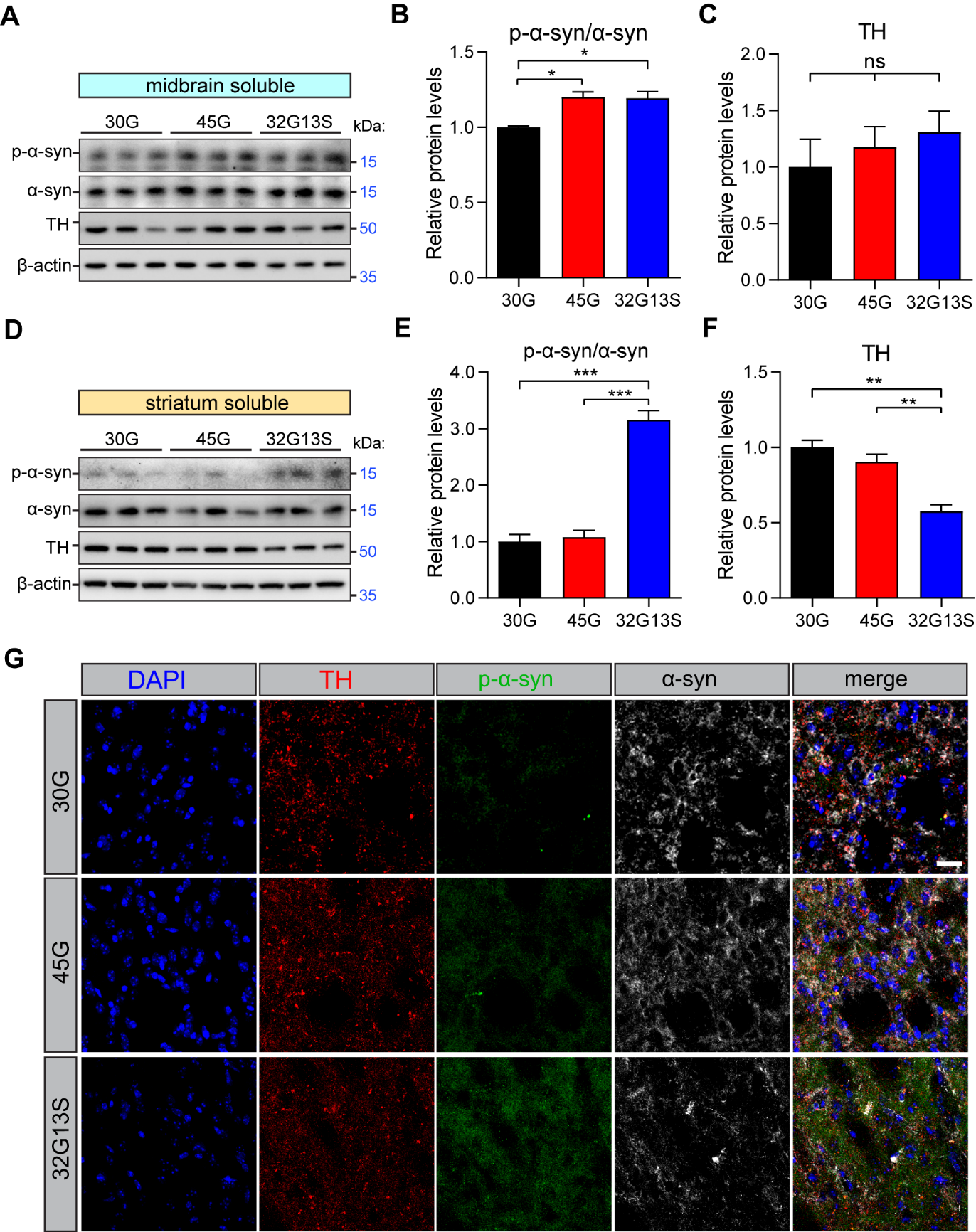


**Supplementary Fig. 3 N2C-polyG intermediate repeat with serine exhibits early PD-like phenotypes. (A)** Western blot of 12-month-old N2C-30G, -45G, -32G13S mouse midbrain soluble proteins. (**B-C**) Quantification of p-α-synuclein (S129) to α-synuclein (**B**) and TH (**C**) in **A**. **(D)** Western blot of 12-month-old N2C-30G, -45G, -32G13S mouse striatum soluble proteins. (**E-F**) Quantification of p-α-synuclein (S129) to α-synuclein (**E**) and TH (**F**) in **D**. (**G**) Immunohistochemical staining of 12-month-old N2C-30G, -45G, -32G13S mouse striatum region by TH (red), p-α-synuclein (S129) (green), and α-synuclein (S129) (grey). The scale bar in the left overview panel is 200 µm. The scale bar in the zoomed picture is 20 µm. Data are presented as the mean ± SD; n = 3 per group. Ns = no significance, **P* < 0.05, ***P* < 0.01, and ****P* < 0.001 by one-way ANOVA with Tukey’s post *hoc* test.

**Supplementary Figure 4**


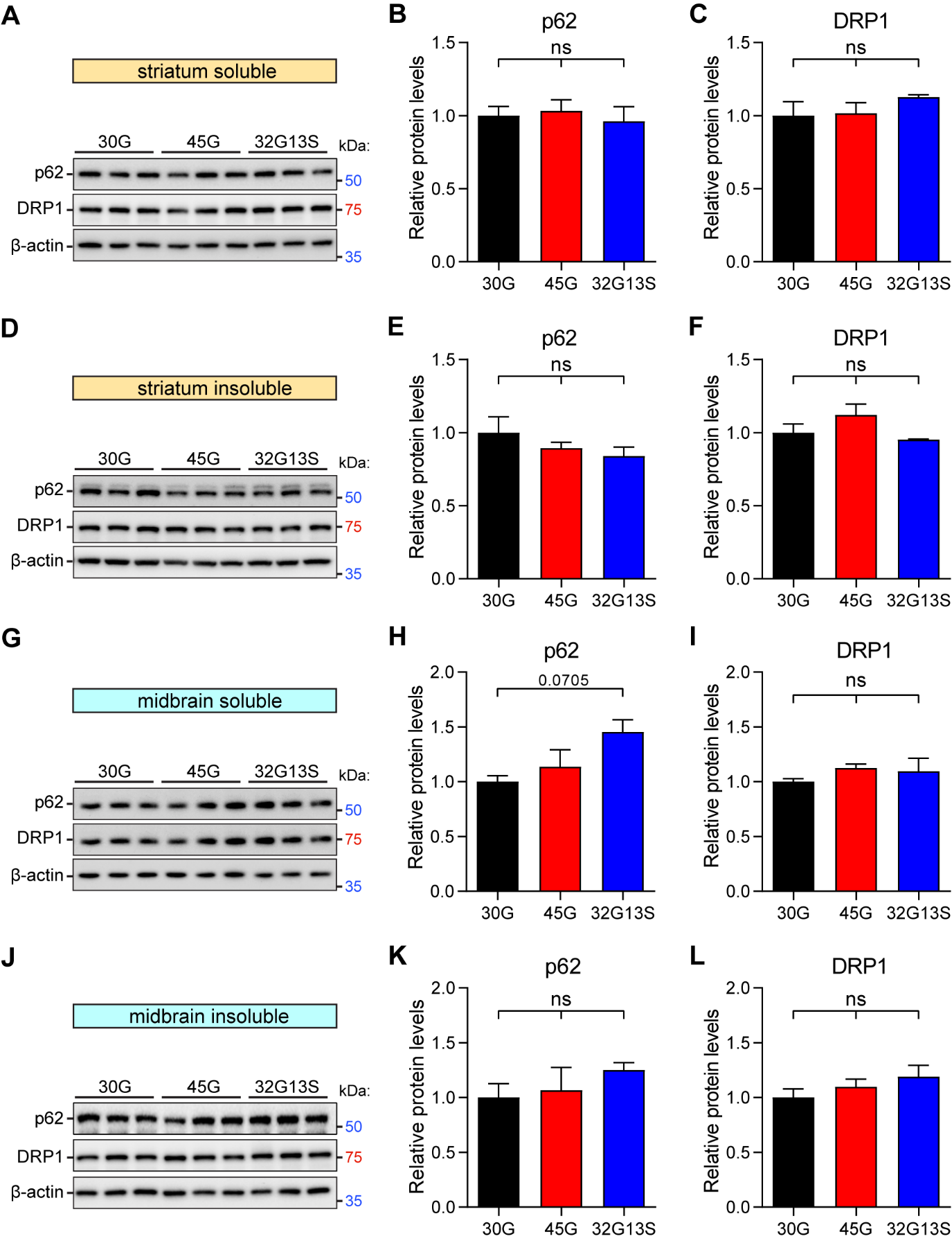


**Supplementary Fig. 4 No mitochondrial dysfunction is detected in the striatum and midbrain of N2C-polyG mice.** Western blot images of 12-month-old N2C-30G, -45G, -and 32G13S mouse soluble (**A**) and insoluble (**D**) striatum proteins, as well as soluble (**G**) and insoluble (**J**) midbrain proteins. Quantification of p62 (**B**, **E**, **H**, and **K**) and DRP1 (**C**, **F**, **I**, and **L**) protein levels in **A**, **D**, **G**, and **J,** respectively. Data are presented as the mean ± SD; n = 3 per group. Ns = no significance by one-way ANOVA with Tukey’s post *hoc* test.

**Supplementary Figure 5**


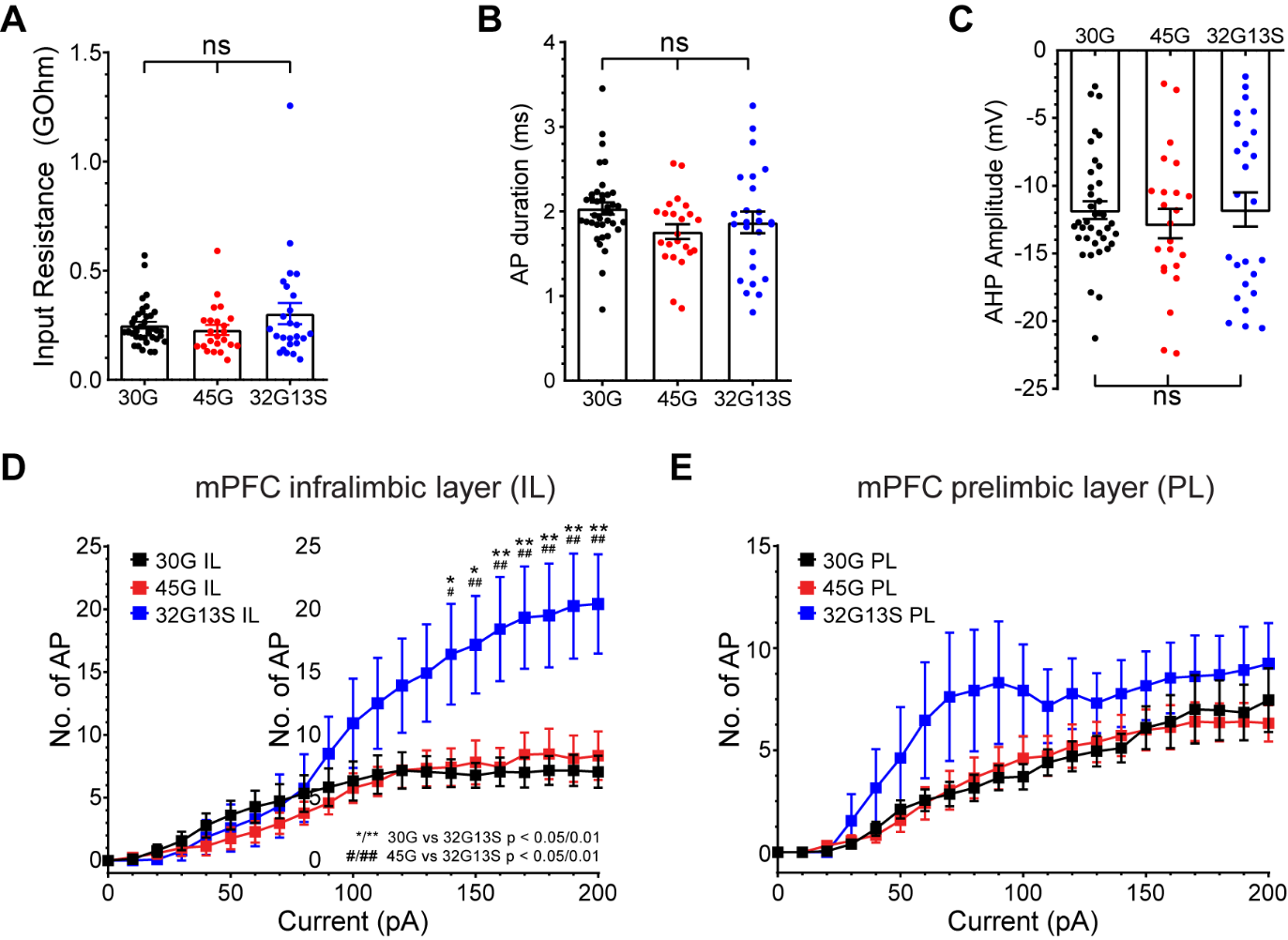


**Supplementary** **Fig. 5 N2C-polyG intermediate with serine insertion induces neuronal hyperexcitability in transgenic mice.** 16-month-old N2C-30G, -45G, or -32G13S transgenic mouse prefrontal cortex cells were used for electrophysiological tests. (**A**) Input resistance (Rin), (**B**) AP duration, and (**C**) AHP amplitude of neurons with N2C-30G, -45G, or -32G13S. N numbers: 30G = 38, 45G = 23, 32G13S = 25. Three independent mice per genotype were used for data collection. One-way ANOVA with Tukey’s post *hoc* test was used for statistical analysis. Data are presented as the mean ± SEM. (**D-E**) Prefrontal cells were further divided into infralimbic layer (IL) and prelimbic layer (PL). The numbers of AP are shown in IL (**D**) and PL (**E**). * represents comparisons between 32G13S and 30G. # represents comparisons between 32G13S and 45G. **IL**: 30G n = 18, 45G = 21, 32G13S = 12. **PL**: 30G = 20, 45G = 25, 32G13S = 13. One-way ANOVA with Tukey’s post *hoc* test was used for statistical analysis. Data are presented as the mean ± SEM. **P* < 0.05 and ***P* < 0.01.

**Supplementary Figure 6**


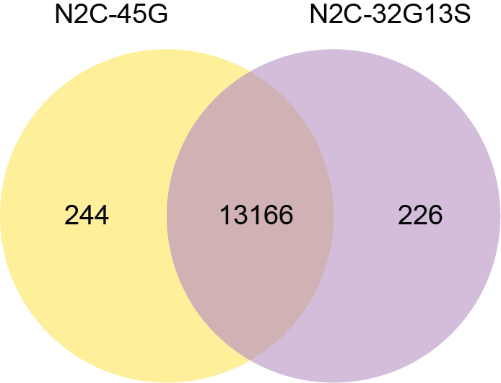


**Supplementary Fig. 6 Differentially expressed genes in N2C-32G13S vs N2C-45G mouse cortex**. Cortex samples from 12-month-old mice were used for bulk RNA sequencing. n = 3 mice per group. Coexpression Venn diagram showing the genes detected in N2C-45G and N2C-32G13S mouse groups. There are 13,166 common differentially expressed genes between N2C-32G13S and N2C-45G, while 244 genes are unique to N2C-45G and 226 genes are unique to N2C-32G13S.

**Supplementary Figure 7**


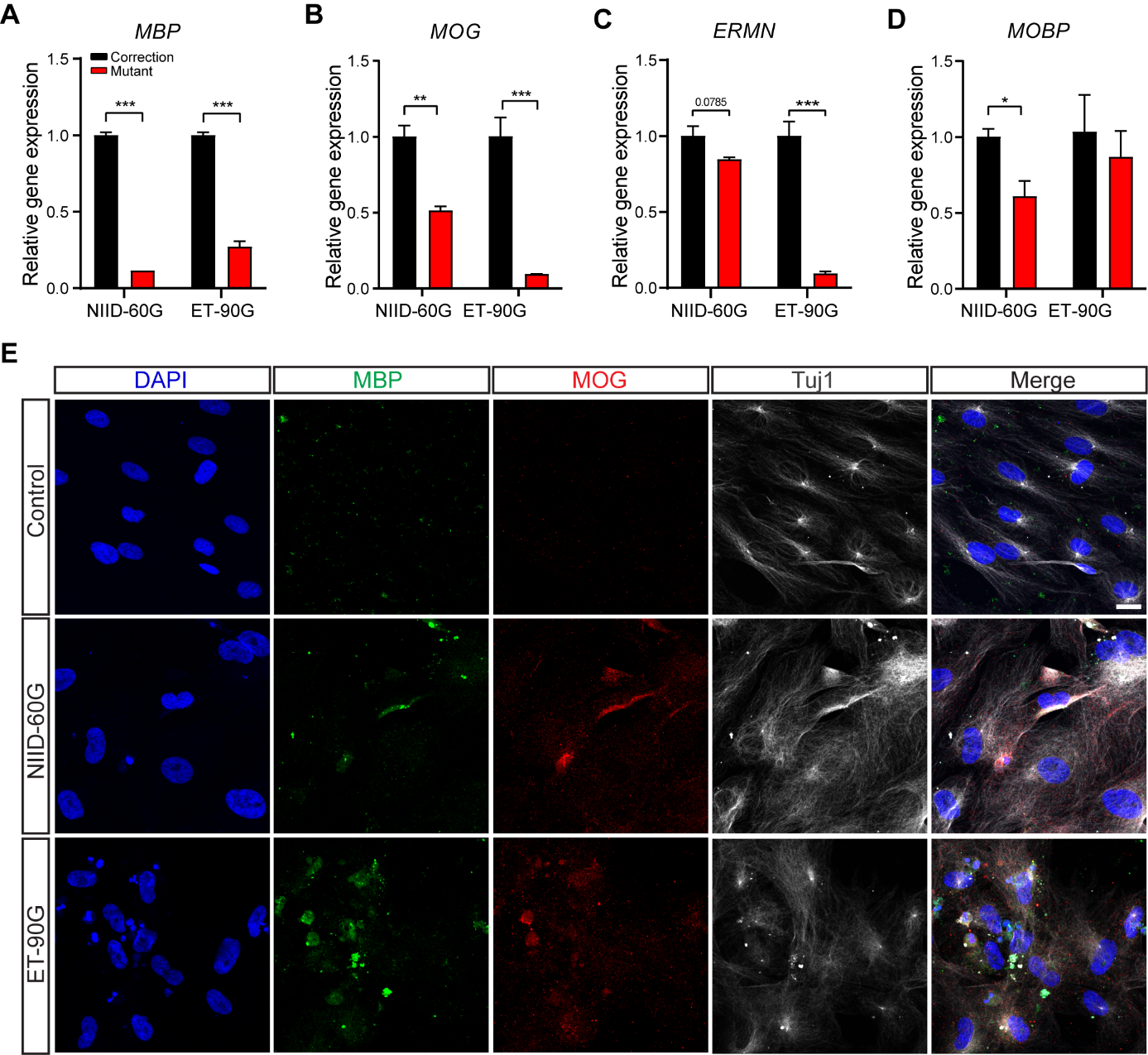


**Supplementary Fig. 7 Myelin sheath component genes are dysregulated in patient iPSC-derived neurons.** IPSCs-differentiated neurons from an NIID patient with 60 GGC repeats and an ET patient with 90 GGC repeats were used for qPCR and immunocytochemical staining. CRISPR-Cas9 genome-corrected iPSC-differentiated neurons were used as their respective controls. **(A-D)** Quantification of relative mRNA levels for *MBP* (**A**), *MOG* (*B*), *ERMN* (**C**), and *MOBP* (**D**) from qPCR. Data are presented as the mean ± SD, n = 3. **P* < 0.05, ***P* < 0.01, and ****P* < 0.001 by two-way Student’s *t* test. (**E**) Representative images of immunocytochemical staining of patient iPSCs-derived neurons by MBP (green), MOG (red), and Tuj1 (grey) antibodies. Scale bar = 20 µm.

**Supplementary Figure 8**


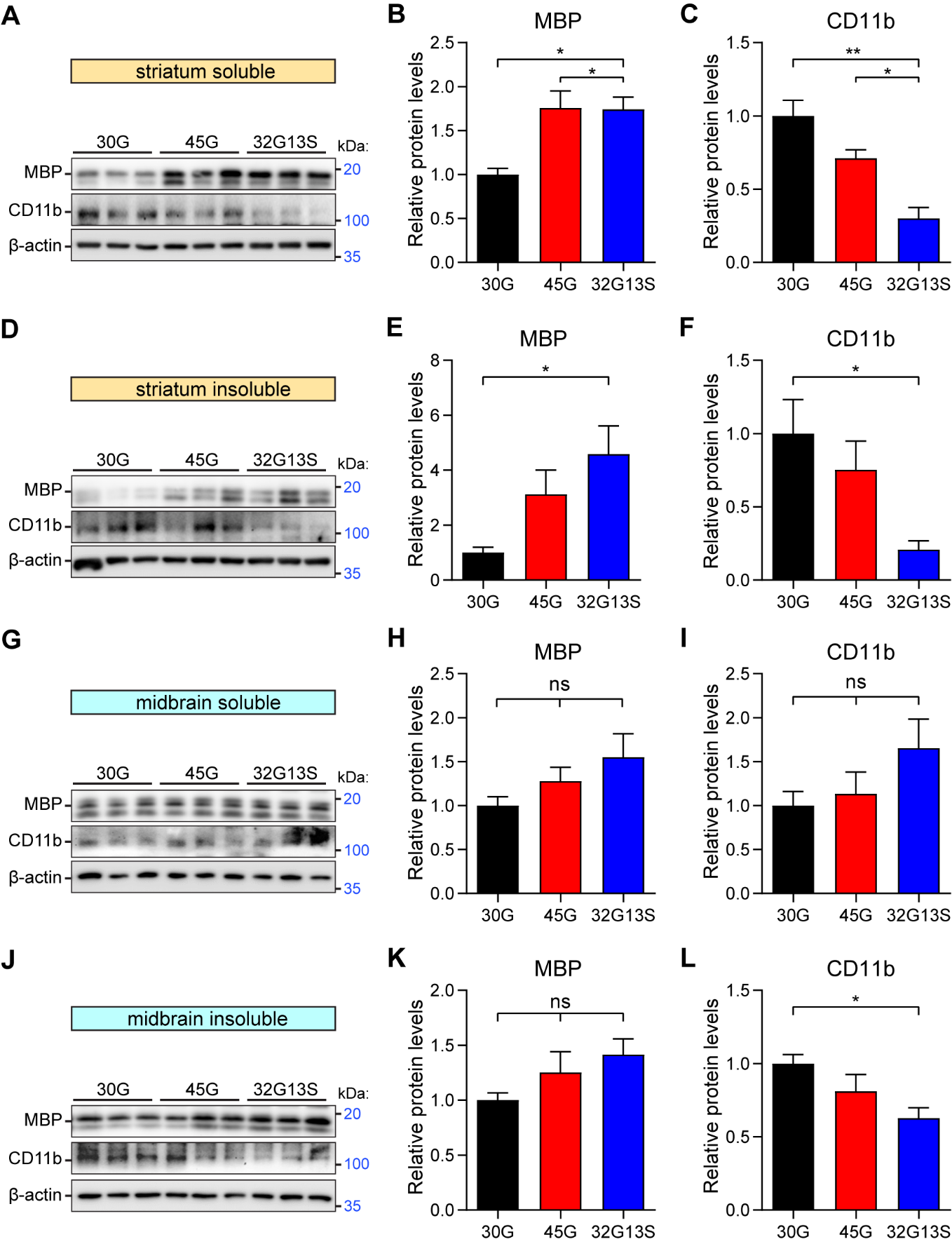


**Supplementary Fig. 8 Hypermyelination in striatum and reduced microglia in both striatum and midbrain of 32G13S mice.** Western blot analysis of soluble (A) and insoluble (D) striatum proteins, as well as soluble (G) and insoluble (J) midbrain proteins, from 12-month-old N2C-30G, -45G, and -32G13S mice. Quantification of MBP (**B**, **E**, **H**, and **K**) and CD11b (**C**, **F**, **I**, and **L**) protein levels in **A**, **D**, **G**, and **J,** respectively. Data are presented as the mean ± SD; n = 3 mice per group. Ns = no significance. **P* < 0.05 and ***P* < 0.01 by one-way ANOVA with Tukey’s post *hoc* test.

**Supplementary Table 1.** **Summary of phenotypes of current *NOTCH2NLC* study models**

| **Models** | **References** | **Methods** | **Sizes** | **Phenotypes and main findings** |
| --- | --- | --- | --- | --- |
| **Drosophila** | Yu et al.,  *PNAS*,  2022 [1] | Transgenic | 100G | - Progressive retinal degeneration in fly eyes - Locomotor impairment and reduced lifespan - Mitochondrial swelling and downregulated mitochondrial complex genes |
| **Mouse** | Boivin et al., *Neuron*,  2021 [2] | AAV-injection | 100G | - Pathogenic expression of uN2CpolyG in animals - Reduced lifespan in uN2CpolyG mice - Motor behavior impairment induced by uN2CpolyG in mice |
|  | Zhong et al.,  *Acta Neuropath.* 2021 [3] | In‑utero electroporation | 70G | - Typical NIID pathologies with p62/ubiquitin/SUMO1-positive intranuclear inclusions |
|  | Liu et al.,  *Sci. Adv*,  2022 [4] | Transgenic | 98G | - Inclusions mainly in the striatum, hippocampus (CA1) and cortex - Early mortality in *NOTCH2NLC*-polyG mice - Neurodegeneration and muscle degeneration in mice - Behavioral phenotypes of locomotor deficits and dementia |
|  | Pan et al., *Cell Biosci.*, 2023 [5] |  | 100G | - Cardiac dysfunction caused by *NOTCH2NLC*-polyG - Mitochondrial dysfunction in cardiac myocytes caused by *NOTCH2NLC*-polyG |
|  | Zhong et al.,  *Acta Neuropath.* 2024 [6] | AAV-injection | 100G | - Impaired motor and cognitive performance - Shortened lifespan and pathologic lesions (including white-matter lesions, microgliosis, and astrogliosis) - Contribution of microglia to polyG-dependent neurodegeneration - Demyelination in the cerebellum |
| **iPSCs-derived cerebral organoids** | Fan et al.,  *Brain*,  2023 [7] | NIID Patients | 90G, 93G, 139G | - Intranuclear inclusions in D90 NIID organoids and D30 neurons - Increased autophagic flux and activation of the integrated stress response - Impact on ribosome biogenesis and translation - Induction of nucleolar stress and impairment of ribosome biogenesis |

**Supplementary Table 2. Dysregulated myelin sheath gene list in N2C-32G13S vs. N2C-45G mice**

| **Genes** | **padj** | **Fold change** | **Gene description** |
| --- | --- | --- | --- |
| ***CLDN11*** | 1.41E-14 | 0.673 | claudin 11 |
| ***GJC2*** | 7.11E-08 | 0.623 | gap junction protein, gamma 2 |
| ***MOG*** | 9.97E-11 | 0.659 | myelin oligodendrocyte glycoprotein |
| ***MOBP*** | 9.50E-24 | 0.665 | myelin-associated oligodendrocytic basic protein |
| ***GNB4*** | 0.002443 | 1.702 | guanine nucleotide binding protein (G protein), beta 4 |
| ***ERMN*** | 1.67E-08 | 0.662 | ermin, ERM-like protein |
| ***MBP*** | 2.73E-05 | 0.718 | myelin basic protein |

The gene list was generated by the GO term analysis using padj < 0.01 and Fc < 0.67 or > 1.5 except *MBP*, which is a core myelin sheath component and added in after GO analysis. The GO term analysis dataset was from bulk RNA sequencing of the cortex from 12-month-old mice.

**Supplementary Table 3.** **Summary of biochemical and pathophysiological changes in 32G13S mice**

| **Markers** | **Midbrain (SNpc)** | **Striatum** | **Cortex** |
| --- | --- | --- | --- |
| **α-synuclein aggregation** | ↑↑↑ (fiber) | ↑↑ | N.S. |
| **Tyrosine Hydroxylase (TH)** | N.S. | ↓ | ↓ |
| **N2C aggregation** | N.S. | N.S. | ↑↑ |
| **Mitochondrial function** | N.S. | N.S. | ↓↓ |

N.S. = No significance. SNpc = Substantia nigra pars compacta.

**References**

1. Yu J, Liufu T, Zheng Y, Xu J, Meng L, Zhang W, Yuan Y, Hong D, Charlet-Berguerand N, Wang Z, Deng J: **CGG repeat expansion in NOTCH2NLC causes mitochondrial dysfunction and progressive neurodegeneration in Drosophila model.** *Proc Natl Acad Sci U S A* 2022, **119:**e2208649119.

2. Boivin M, Deng J, Pfister V, Grandgirard E, Oulad-Abdelghani M, Morlet B, Ruffenach F, Negroni L, Koebel P, Jacob H, et al: **Translation of GGC repeat expansions into a toxic polyglycine protein in NIID defines a novel class of human genetic disorders: The polyG diseases.** *Neuron* 2021, **109:**1825-1835 e1825.

3. Zhong S, Lian Y, Luo W, Luo R, Wu X, Ji J, Ji Y, Ding J, Wang X: **Upstream open reading frame with NOTCH2NLC GGC expansion generates polyglycine aggregates and disrupts nucleocytoplasmic transport: implications for polyglycine diseases.** *Acta Neuropathol* 2021, **142:**1003-1023.

4. Liu Q, Zhang K, Kang Y, Li Y, Deng P, Li Y, Tian Y, Sun Q, Tang Y, Xu K, et al: **Expression of expanded GGC repeats within NOTCH2NLC causes behavioral deficits and neurodegeneration in a mouse model of neuronal intranuclear inclusion disease.** *Sci Adv* 2022, **8:**eadd6391.

5. Pan Y, Jiang Y, Wan J, Hu Z, Jiang H, Shen L, Tang B, Tian Y, Liu Q: **Expression of expanded GGC repeats within NOTCH2NLC causes cardiac dysfunction in mouse models.** *Cell Biosci* 2023, **13:**157.

6. Zhong S, Lian Y, Zhou B, Ren R, Duan L, Pan Y, Gong Y, Wu X, Cheng D, Zhang P, et al: **Microglia contribute to polyG-dependent neurodegeneration in neuronal intranuclear inclusion disease.** *Acta Neuropathol* 2024, **148:**21.

7. Fan Y, Li MJ, Yang J, Li SJ, Hao XY, Li JD, Wang YC, Tang MB, Zhang C, Shi JJ, et al: **GGC repeat expansion in NOTCH2NLC induces dysfunction in ribosome biogenesis and translation.** *Brain* 2023, **146:**3373-3391.
